# Selective Targeting of Regulated Rhabdomyosarcoma Cells by Trinuclear Ruthenium(II)–Arene Complexes

**DOI:** 10.1021/acs.jmedchem.4c00256

**Published:** 2024-04-03

**Authors:** Athi Welsh, Karabo Serala, Sharon Prince, Gregory S. Smith

**Affiliations:** † Department of Chemistry, University of Cape Town, Rondebosch, Cape Town 7700, South Africa; ‡ Department of Human Biology, Faculty of Health Science, University of Cape Town, Observatory, Cape Town 7935, South Africa

## Abstract

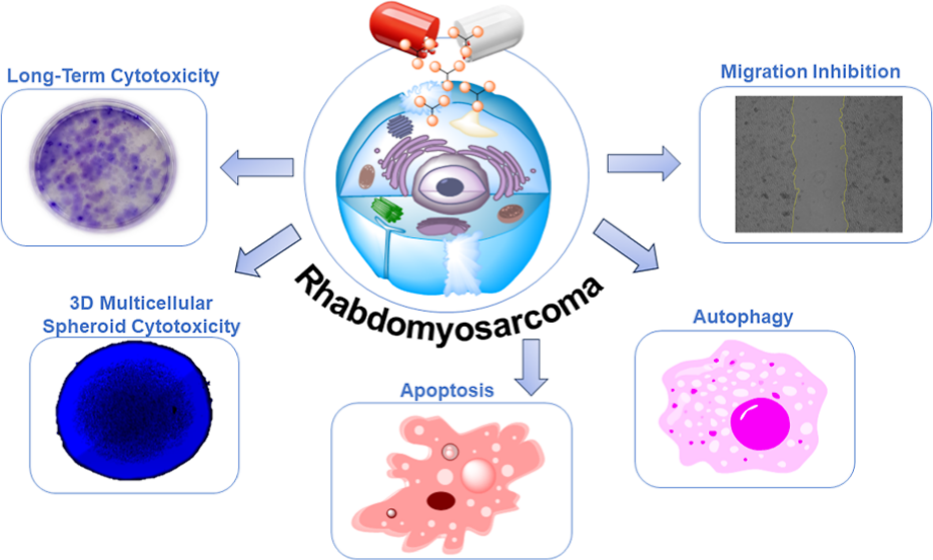

The use of benzimidazole-based trinuclear ruthenium(II)–arene complexes (**1**–**3**) to selectively target the rare cancer rhabdomyosarcoma is reported. Preliminary cytotoxic evaluations of the ruthenium complexes in an eight-cancer cell line panel revealed enhanced, selective cytotoxicity toward rhabdomyosarcoma cells (RMS). The trinuclear complex **1** was noted to show superior short- and long-term cytotoxicity in RMS cell lines and enhanced selectivity relative to cisplatin. Remarkably, **1** inhibits the migration of metastatic RMS cells and maintains superior activity in a 3D multicellular spheroid model in comparison to that of the clinically used cisplatin. Mechanistic insights reveal that **1** effectively induces genomic DNA damage, initiates autophagy, and prompts the intrinsic and extrinsic apoptotic pathways in RMS cells. To the best of our knowledge, **1** is the first trinuclear ruthenium(II) arene complex to selectively kill RMS cells in 2D and 3D cell cultures.

## 1 Introduction

Despite significant research efforts and investment over the past 20 years, cancer remains a leading cause of death with over 19.3 million new cases and 10 million deaths reported globally in 2020.^1,2^ One of the most commonly used treatment modalities involves the use of chemotherapeutics, with platinum metallodrugs cisplatin, carboplatin, and oxaliplatin demonstrating global clinical success and being frequently utilized as frontline metal-based therapeutics with FDA approval.^3^ At least half of all cancer patients are estimated to receive these nascent platinum-based chemotherapies at some point during their treatment regimens, despite the unfavorable side effects that are frequently linked to their use (such as nephrotoxicity, myelosuppression, and chemotherapy-induced nausea and vomiting), as well as the growing number of platinum-resistant cancers.^4−8^ To circumvent these detrimental attributes associated with the archetypical platinum metallodrugs, several alternative strategies have been explored including the development of metallodrugs with alternate platinum-group metals (PGMs), the development of multinuclear complexes, and more recently, the investigation of heteronuclear metal complexes and nanotechnology.^9−13^

Ruthenium is currently at the forefront of PGM-based metallodrug discovery. This is due to the favorable biological characteristics associated with ruthenium-containing complexes including selective uptake, enhanced selectivity relative to platinum metallodrugs, and the various oxidation states that are accessible *in vitro*.^14,15^ Moreover, there are several reports of ruthenium-based metal complexes that exert cytotoxic effects through a range of mechanisms of action including apoptosis induction, glucose metabolism reprogramming, HER-2 repression, necroptosis induction by targeting topoisomerases I and II, and induction of redox imbalance, all of which provide an impetus for the development of ruthenium-based cytotoxic agents.^16−21^

Polymetallic complexes are complexes that comprise of more than one metal center. Generally, polymetallic complexes have been reported to show enhanced cytotoxicity and novel mechanisms of action relative to their mononuclear counterparts, which provides further incentive to the investigation of these types of complexes as anticancer agents.^22−25^ Encouraged by the discovery of the trinuclear complex [*trans*-diamminechloroplatinum(II)] [μ-*trans*-diamminebis(hexanediamine)platinum(II)] nitrate (**BBR3464**) as the first polynuclear platinum drug to enter clinical trials, interest in the application of other polymetallic complexes as chemotherapeutic agents has grown substantially in the last two decades.^25−31^ Despite the poor response rates and high toxicity of trimetallic **BBR3464**, the field of trinuclear metallodrug discovery remains relatively unexplored, highlighting the niche potential of polymetallic drugs in cancer therapy.

The use of *N*-heterocycles as nitrogen-donor ligands in the synthesis of ruthenium-based complexes has proven to be quite successful in metal-based drug discovery, and is exemplified by complexes such as **BOLD-100** and **NAMI-A,** which have made it to various phases of clinical trials.^32−34^ The benzimidazole scaffold is one of the top 10 nitrogen heterocycles among FDA-approved drugs and remains a mainstay in the development of chemotherapeutic agents.^35,36^ Investigation of the benzimidazole pharmacophore as a constituent of novel anticancer drug candidates is further promulgated by a wide range of mechanisms of action that benzimidazole-based compounds elicit, including repression of KRAS and ROS-JNK pathways, inducing mitotic catastrophe, and androgen receptor antagonism.^37−41^ Combining a metal center with pharmacologically privileged scaffolds, such as the benzimidazole heterocycle, paves the way for the development of novel chemotherapeutics, as a synergism is commonly observed in the resulting organometallic complex.^42−45^ It is on this premise that this work is based, in which a series of trinuclear 2-quinolylbenzimidazole-based ruthenium(II) complexes containing different arene ligands were evaluated for biological activity in a panel of 2D cancer cell lines and 3D cancer spheroid models. We show for the first time that one of these trinuclear complexes exhibit potent and selective short- and long term cytotoxicity and antimigratory effects in 2D and 3D rhabdomyscarcoma cell culture models. This is the first study that reveals a trinuclear complex as a potential chemotherapeutic treatment for rhabdomyosarcoma (RMS).

## 2 Results and Discussion

### 2.1 Synthesis and Characterization of Trimetallic Ruthenium(II) Complexes (**1–3**)

**Scheme 1:**
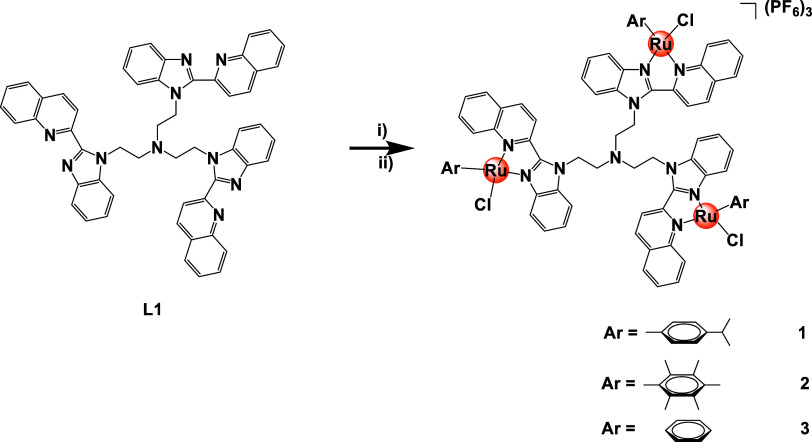
Reagents and conditions: (i) DCM: EtOH (1:1)/[Ru(*p*-cymene)Cl_2_]_2_, [Ru(hexamethylbenzene)Cl_2_]_2_, [Ru(benzene)Cl_2_]_2_/RT of 40 °C/24 or 48 h. (ii) DCM: EtOH (v/v 1:1)/NH_4_PF_6_/RT/1 h.

The trinuclear ruthenium(II) arene complexes (**1**–**3**) were synthesized via two steps: (i) the reaction of the tris-2-quinolylbenzimidazole ligand (**L1**), synthesized using procedures previously reported in the literature,^46^ with either [Ru(*p*-cymene)Cl_2_]_2_, [Ru(hexamethylbenzene)Cl_2_]_2_ or [Ru(benzene)Cl_2_]_2_, and (ii) a salt metathesis reaction with ammonium hexafluorophosphate (Scheme 1). All of the ruthenium(II) complexes were isolated as hexafluorophosphate salts in excellent yields (95–98%). The organoruthenium(II) complexes were fully characterized using ^1^H, ^13^C, and ^31^P NMR, UV–vis and infrared spectroscopy, ESI mass spectrometry, and HPLC (purity >95%), as shown in Figures S1–S9 in the Supporting Information.

The assessment of the aqueous stability of compounds intended for biological evaluation is a vital parameter in the identification of viable drug leads.^47^ Generally, these assessments are conducted in aqueous media with an appreciable amount of DMSO, which is one of the most widely used organic solvents in the biological screening of potential cytotoxic agents.^48,49^ With this in mind, the stability of the triruthenium(II) complexes was evaluated in a 1:99 DMSO/cell culture growth media (RPMI-1640) mixture and monitored using UV/vis spectroscopy at 37 °C over 48 h.

Generally, there were no significant changes noted in the UV/vis spectra of complexes **1**–**3** over the duration of the study (Figure S10 in Supporting Information). This suggests that the trinuclear complexes maintain their structural integrity in growth media and the complexes do not react with DMSO nor the components of the cell culture media.^50^ As a result, the trinuclear complexes were deemed to be suitable for biological testing.

### 2.2 *In Vitro* Prescreening of the Trimetallic Ruthenium(II) Complexes (**1–3**)

To identify the most active ruthenium(II) complex, the aforementioned complexes (**1**–**3**) were evaluated for their short-term cytotoxicity in a panel of eight different cancer cell lines at two fixed concentrations (10 and 20 μM) using the MTT (3-(4,5-dimethylthiazol-2-yl)-2,5 diphenyltetrazolium bromide) assay.^51^ The initial concentration was selected based on the approach by the National Cancer Institute (NCI, USA), which conducts a preliminary screening of potential anticancer drug candidates at 10 μM.^52,53^ The organoruthenium(II) complexes were preliminarily evaluated for their cytotoxicity in breast (MCF-7 and MDA-MB-231), RMS (RD and RH-30), pancreatic (PANC-1 and CFPAC-1), and cervical (HeLa and Caski) cancer cell lines. These types of cancers were selected, as they have a high diagnosis rate and account for a high number of cancer-related mortalities.^1,54−57^

A general cytotoxicity trend can be observed by comparing the cytotoxicities of complexes (**1**–**3**) among the four distinct types of cancers: RMS > pancreatic cancer > breast cancer > cervical cancer. These findings emphasize the importance of screening potential drug leads against a variety of cancers, as different types of cancers show varying sensitivities to the tested trimetallic complexes. Furthermore, it is worth noting that two of the three complexes (**1** and **2**) show activity that is either comparable or more potent relative to cisplatin in the breast cancer, pancreatic cancer, and RMS cell lines at fixed concentrations (10 and 20 μM).

Generally, the trinuclear ruthenium(II) arene complexes were noted to show dose-dependent cytotoxicity, with a significant increase in the cytotoxic activity of all the complexes upon doubling the treatment concentration from 10 to 20 μM (Figures S11–S18, in the Supporting Information). The complexes (**1–3**) were also noted to show the following trend in their cytotoxicity across all the tested cell lines, **1** > **2** > **3**, with the trinuclear complex containing the *p*-cymene ancillary ligand (**1**) showing the best cytotoxicity at fixed concentrations (10 and 20 μM). Although these are preliminary cytotoxicity screenings, the important role that the ancillary arene ligand plays in influencing the overall biological activity of the trinuclear complexes is highlighted. As previously shown, the cytotoxicity of these benzimidazole-based ruthenium(II) complexes (**1–3**) is highly dependent on the nature of the arene ancillary ligand, with the trinuclear complex **1** containing the *p*-cymene ligand showing activity superior to that of the other tested complexes (**2** and **3**). This phenomenon is well documented in the literature with complexes containing the *p*-cymene ligand generally shown to be more active.^58−62^

Taken together, the preliminary screening results show that complexes **1** and **2**, bearing the ancillary ligands *p*-cymene and hexamethylbenzene, respectively, show promising cytotoxicity in RMS, pancreatic cancer, and breast cancer cell lines. Consequently, these complexes (**1** and **2**) were selected for multidose studies to determine their half maximal inhibitory concentrations (IC_50_ concentrations) in the aforementioned cancer cell lines.

### 2.3 *In Vitro* Multidose Screening of Selected Trinuclear Ruthenium(II) Complexes (**1** and **2**) in Selected Cell Lines

To determine the IC_50_ concentrations of complexes **1** and **2**, cancer cells were treated with varying concentrations of the chosen complexes, with cisplatin serving as both a positive control and a benchmark.

In the MCF-7 breast cancer cell line (data summarized in Table 1), the *p*-cymene containing ruthenium(II) complex **1** showed enhanced cytotoxicity relative to that of **2**. This observation is consistent with that noted in the preliminary screening of **1** in the MCF-7 cell line. Generally, the cytotoxicity of **1** is greater than that of complex **2**, which is also consistent with the prescreen data. In the triple-negative MDA-MB-231 breast cancer cell line, a similar general trend in the cytotoxicity of complexes **1** and **2** was delineated (Table 1). This pattern supports the prescreen data once more. However, in the MDA-MB-231 cell line, neither **1** nor **2** exhibits comparable or enhanced cytotoxicity relative to cisplatin.

**Table 1:** In Vitro Cytotoxicity of the Selected Ruthenium(II) Arene Complexes (**1** and **2**) Represented as Half-Maximal Inhibitory Concentrations (IC_50_) in RMS, Breast, and Pancreatic Cancer Cell Lines, and Non-tumorigenic Human Fibroblast Cells (FG-0)

cell line	cell type	IC_50_ ± SEM (μM)			S.I. (FG-0)^b^		
		**1**	**2**	cisplatin	**1**	**2**	cisplatin
RD	RMS	17.64 ± 0.59	54.17 ± 4.02^a^	29.51 ± 1.46	2.05	0.90	1.48
RH-30	RMS	17.02 ± 0.68	50.77 ± 2.89^a^	36.73 ± 1.03	2.12	0.96	1.18
MCF-7	breast cancer	23.54 ± 1.48	32.92 ± 1.05	24.92 ± 1.03	1.53	1.48	1.75
MDA-MB-231	breast cancer	30.20 ± 1.43	39.38 ± 1.44^a^	24.45 ± 1.03	1.19	1.24	1.78
PANC-1	pancreatic cancer	19.28 ± 1.16		31.08 ± 1.044	1.88		1.40
CFPAC-1	pancreatic cancer	26.71 ± 1.05		18.85 ± 1.07	1.35		2.31
FG-0	human dermal fibroblasts	36.19 ± 1.05^a^	48.88 ± 1.15^a^	43.52 ± 1.08^a^			

a IC_50_ value extrapolated by Graphpad Prism V8.0.2.

b S.I.: Selectivity index (IC_50_ FG-0/IC_50_ Cancerous Cell line).

From the results obtained in the pancreatic cancer (PANC-1) cell lines (Table 1), **1** is noted to show enhanced biological activity. However, in the highly metastatic CFPAC-1 pancreatic cancer cell line, **1** is less cytotoxic, relative to cisplatin. These findings corroborate the prescreen studies in which cisplatin was more cytotoxic relative to complex **1** in the CFPAC-1 pancreatic cancer cell line.

Complex **1** is the most cytotoxic complex in both RMS cell lines (RD and RH-30) and exhibits enhanced cytotoxicity relative to clinically used cisplatin in both RMS cell lines, which again is consistent with the prescreen data. In the case of the trinuclear ruthenium(II) complex containing the hexamethylbenzene ancillary ligand (**2**), the complex did not reduce cell viability to ≤50%. As such, the IC_50_ of **2** was not experimentally determined and was extrapolated from the experimental data using the GraphPad Prism software and found to be 54.17 and 50.77 μM in the RD and RH-30 cell lines, respectively.

The cytotoxicity of the selected trinuclear ruthenium(II) complexes **1** and **2** was also evaluated in the nontumorigenic human dermal fibroblast (FG-0) cell line. Generally, **1** and **2** were found to have milder cytotoxicity in the nontumorigenic FG-0 cell line in comparison with the malignant cell lines investigated in this study (Table 1), indicating that **1** and **2** have appreciable cytotoxicity toward cancer cells over nontumorigenic cells. Furthermore, **1** was found to be generally more selective than **2**, and more importantly, **1** was found to be more selective than cisplatin. This is supported by **1** having selectivity indices (S.I.) higher than those of both **2** and cisplatin (Table 1). A comparison of the S.I. values of **1** reveal that the complex shows the highest selectivity toward both RMS cell lines (RD and RH-30). Furthermore, because a compound with a selectivity index of 2 is considered highly selective,^63,64^**1** was chosen for further studies in both RMS cell lines.

### 2.4 Long-Term Cytotoxicity and Selectivity of Complex **1** toward RMS Cells

RMS remains the most common soft tissue sarcoma diagnosed in children and while the treatment of localized, primary tumors improves the overall survival rate, chemotherapeutic agents that are commonly used are accompanied by debilitating side effects.^65−67^ Moreover, an excess of 15% of patients are diagnosed with metastatic RMS and there have been limited improvements in the treatment of patients with recurrent and metastatic RMS, with survival rates reportedly between 21 and 30%.^68−71^ Frontline chemotherapeutics used to treat RMS remain unchanged since the late 1970s when VAC, a combination comprising of Vincristine, Actinomycin D, and Cyclophosphamide, was introduced as an effective anticancer drug cocktail to treat RMS.^72^ With this in mind, the potential impact of the trinuclear ruthenium(II) complex **1** on the long-term survival of RMS cells (RD and RH-30) was explored using the clonogenic assay.^73^ Due to the positive response by patients to a combination of cisplatin and the VAC cocktail and the positive response to cisplatin-based treatments, cisplatin was selected as an apt positive control.^74,75^

**Figure 1:**
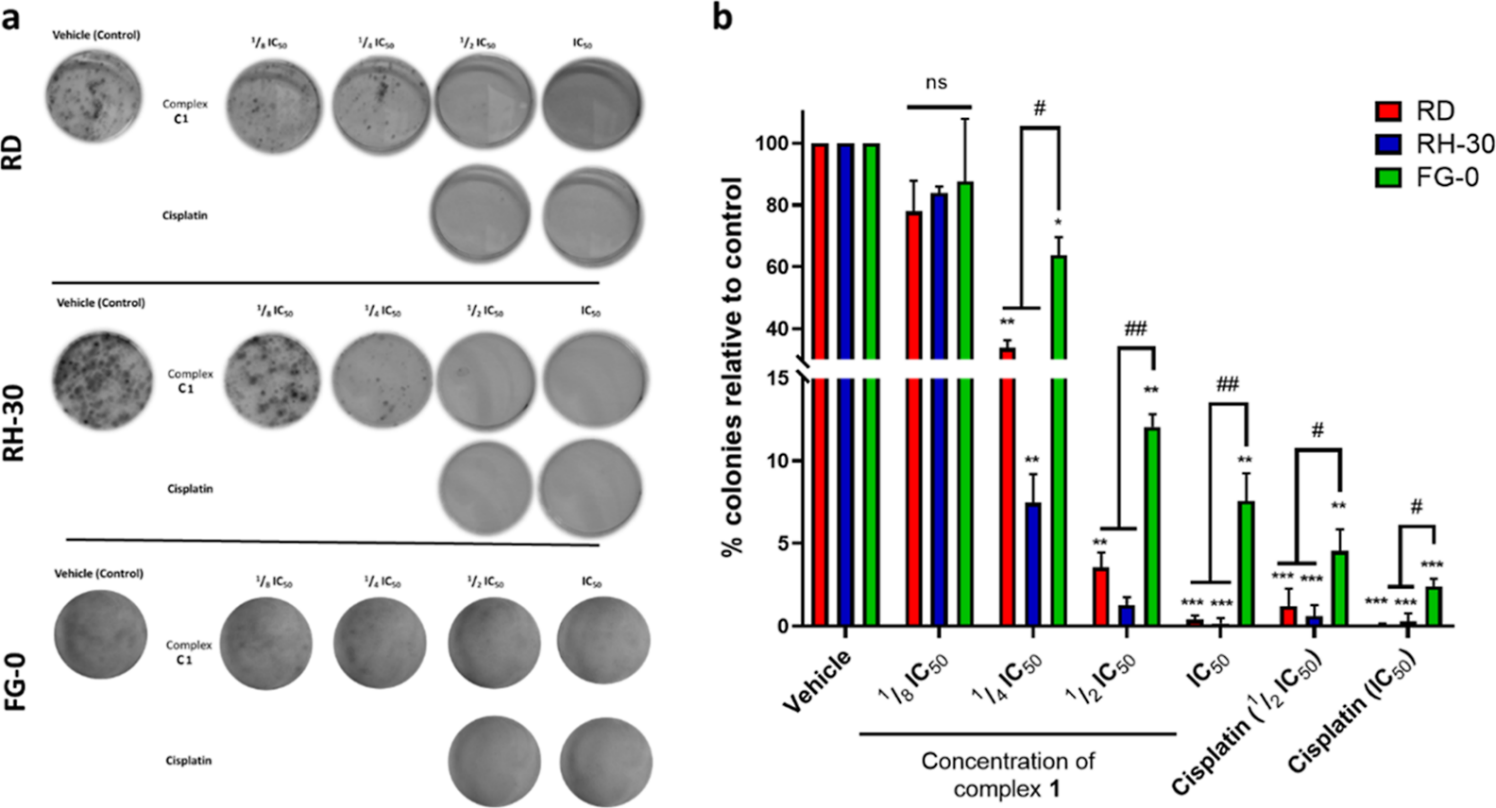
Representative (a) images and (b) quantification of clonogenic assays conducted in the RMS cell lines (RD and RH-30) and the nontumorigenic human fibroblast cell line (FG-0) treated with the vehicle control (0.1% DMSO), ^1^/_8_IC_50_, ^1^/_4_IC_50_, ^1^/_2_IC_50_, and IC_50_ concentrations of **1**. Cisplatin was included as a positive control and a benchmark. Images from three independent repeats were quantified using the Colony Area plugin in ImageJ software. The graphs represent the mean colony area ± SEM of each treatment condition as a percentage of the vehicle control, where ns = not significant,* or #*p* ≤ 0.05, ** or ##*p* ≤ 0.01, and *** or ###*p* ≤ 0.001.

The data obtained for both the RMS cell lines are summarized in Figure 1 and show the representative images (Figure 1a) and the quantification of the colonies formed over the 10–12 day period (Figure 1b). Complex **1** clearly inhibits the survival and colony-forming ability of both RMS cell lines, as the number of colonies in the controls (vehicle) is significantly greater than the colonies formed by RMS cells treated with **1**. Indeed, for both RMS cell lines, there was a significant decrease in the colony area and number after treatment with ^1^/_2_IC_50_ of **1** and no RMS colonies formed after treatment with the IC_50_ concentrations of **1**. It is worth noting that the RMS cells were treated with a significantly lower concentration of **1** relative to cisplatin (the IC_50_ concentration of **1** is 17.65 and 17.02 μM in the RD and RH-30 cell lines, respectively, and the IC_50_ concentration of cisplatin is 29.51 and 36.73 μM in the respective cell lines), suggesting that at a significantly lower concentration, **1** shows appreciable long-term cytotoxicity and may reduce the probability of RMS recurrence. To further investigate the selectivity of **1** toward RMS cancer cells, the long-term cytotoxicity of **1** was evaluated in the nontumorigenic FG-0 cell line. Indeed, **1** reduces FG-0 long-term survival in a dose-dependent manner, despite the dispersed and diffused colonies of the skin fibroblast cell lines due to their physiology.^76^ Overall, **1** is significantly less cytotoxic in nontumorigenic cells (FG-0) relative to cancerous RMS cells (RD and RH-30) at all treated concentrations (excluding at the lowest concentration of **1**,^1^/_8_IC_50_) and is significantly less cytotoxic toward the nontumorigenic cells relative to cisplatin (Figure 1b) at their respective IC_50_ concentrations. This highlights that **1** maintains its superior selectivity relative to cisplatin in the long term, and **1** may have less undesirable side effects relative to clinically used cisplatin.

### 2.5 Antimigratory Behavior of the Trinuclear Ruthenium(II) Benzimidazole-Based Complex (**1**)

Understanding the potential antimetastatic properties of a prospective anticancer drug candidate is critical because metastasis, which is often preceded by migration, is one of the hallmarks of cancer and one of the major challenges in cancer treatment.^77,78^ As a result, the ability of trinuclear complex **1** to inhibit the migration of RMS cells was investigated using scratch motility assays.

**Figure 2:**
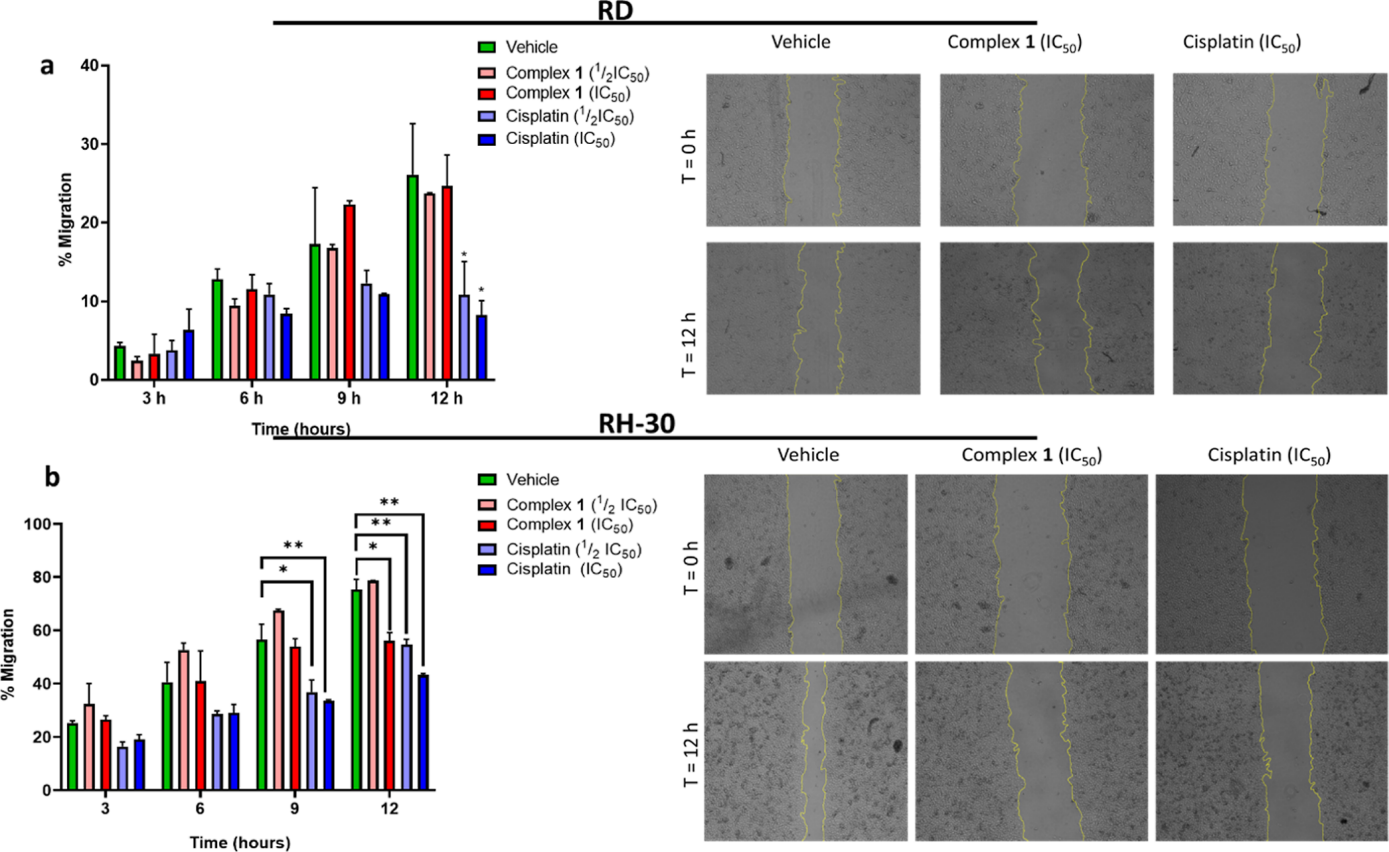
Representative images (10×; EVOS M5000 imaging System) and graphical quantification of migration of (a) RD and (b) RH-30 RMS cell lines treated with either **1** or the vehicle (0.1% DMSO) over 12 h. Cells were photographed at 0, 3, 6, 9, and 12 h post wound formation. Cisplatin was included as a positive control and a benchmark. Images from three independent repeats were quantified using the ImageJ software, and the graphs represent the mean wound area ± SEM of each treatment condition as a percentage of the vehicle control, where *p ≤ 0.05 and **p ≤ 0.01, and thus statistically significant.

In the RD cell line, **1** does not inhibit the migration of RD cells in a statistically significant manner at the tested concentrations (Figure 2a); however, cisplatin only shows significant reduction in the migration of RD cells after 12 h in a dose-independent manner. The statistically insignificant antimigratory activity of **1** or dose-independent activity of cisplatin in the RD cell line is attributed to RD cells being derived from an embryonal RMS tumor (eRMS).^79^ The eRMS subtype of RMS tumors comprises more than 67% of RMS cases. However, these types of tumors are comprised of cells that are less migratory and are localized within the primary tumor site.^80,81^ As a result, the statistically insignificant data obtained for **1** come as no surprise and similar results have been reported by Bleloch *et al*. for **AJ-5**, a binuclear palladacycle, in the RD cell line.^82^

In the more aggressive and metastatic alveolar RMS (aRMS) cells,^65^ the RH-30 cell line, the trinuclear ruthenium(II) complex **1** was noted to inhibit cell migration in a dose-dependent manner after 12 h of treatment (Figure 2b). However, cisplatin was noted to show moderately higher antimigratory activity in the RH-30 cell line. Overall, the benzimidazole-based trinuclear complex **1** shows antimigratory activity in the more aggressive and invasive RH-30 aRMS cell line. Consequently, **1** may inhibit the metastasis of the aRMS subtypes.

### 2.6 Activity of the Triruthenium(II) Complex (**1**) in 3D Cell Culture Models of RMS Cell Lines

The multicellular 3D spheroid culture model has become an essential tool in anticancer drug discovery, as spheroids closely resemble *in vivo* solid tumors in terms of complexity and growth kinetics, offering an efficient method for studying tumor behavior and drug efficacy.^83−85^ Consequently, the effect of **1** on 3D cell culture models in RMS cell lines was investigated.

**Figure 3:**
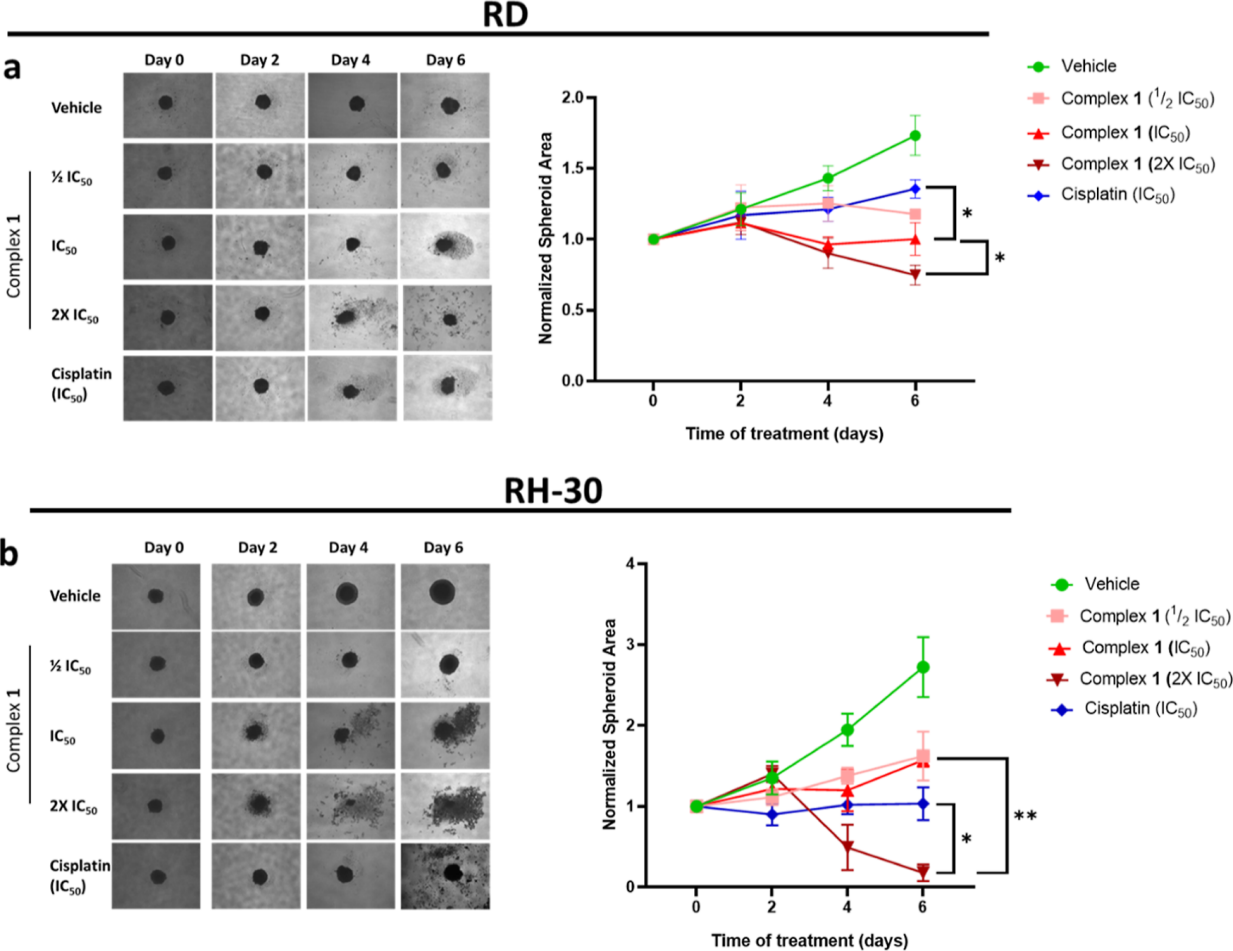
Representative images and quantification of 3D spheroids derived from RMS cell lines (a) RD and (b) RH-30 treated with ^1^/_2_IC_50_, IC_50,_ and 2× IC_50_ concentrations of the trinuclear ruthenium(II) arene complex **1** or cisplatin for 6 days. The area of the spheroids was determined using ImageJ. The graphs represent the mean normalized spheroid size ± SEM for each treatment condition, where *p ≤ 0.05 and **p ≤ 0.01, and thus statistically significant.

In both the RD and RH-30 spheroids, **1** reduced spheroid size in a concentration-dependent manner (Figure 3a,b, respectively). Furthermore, accompanying the significant reduction of size of the spheroids, the number of cells that detached from the spheroid (the “debris” around the spheroid core) increases in a dose-dependent manner over the studied six-day period, suggesting that **1** is indeed impacting the number of viable cells in the 3D spheroid model. The most intriguing observation from the representative spheroid images of the metastatic RH-30 cell line and the graphs in Figure 3b is that on the sixth day of treatment, the spheroids treated with 2× IC_50_ are of negligible size. This implies that **1** may be able to eradicate tumors of the more metastatic RMS subtype.

**Figure 4:**
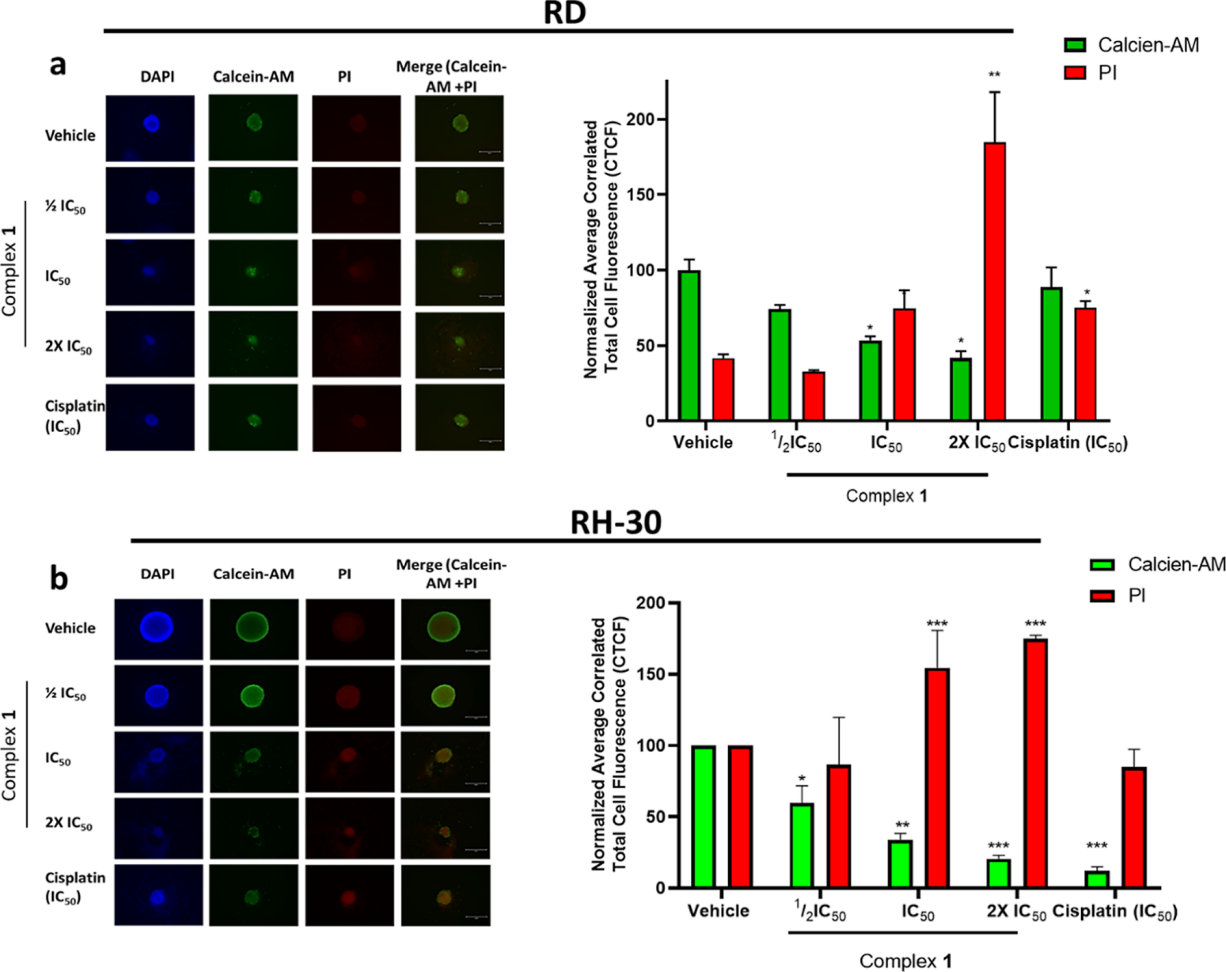
Representative images and quantification of 3D spheroids derived from RMS cell lines (a) RD and (b) RH-30 treated with ^1^/_2_IC_50_, IC_50,_ and 2× IC_50_ concentrations of the trinuclear complex **1** or cisplatin for 6 days, and stained with DAPI, Calcein-AM and propidium iodide (PI). The average correlated total cell fluorescence (CTCF) was determined using ImageJ software. The graphs represent the mean normalized CTCF ±SEM for each treatment condition, where *p ≤ 0.05, **p ≤ 0.01, and ***p ≤ 0.001, thus statistically significant.

To determine and quantify the proportion of cells that remain viable after the treatment of RD and RH-30 spheroids with either the control (vehicle), by varying concentrations of **1,** or cisplatin (at IC_50_), the spheroids were stained with Calcein-AM (green fluorescence) and propidium iodide (PI) (red fluorescence). Calcein-AM passively crosses the cell membrane and is converted by intracellular esterases into a polar, nonmembrane-crossing product (Calcein), which is retained only by viable cells.^86^ The amount of green fluorescence (in the representative images and graphs in Figure 4) is directly proportional to the population of living, viable cells. PI is a DNA-binding molecule that emits red fluorescence and cannot passively enter viable cells with an intact membrane; thus, it can be used to quantify nonviable/dead cells.^87^ The amount of red fluorescence (in the representative images and graphs in Figure 4) is directly proportional to the population of nonviable cells.

Generally, in both the RD and RH-30 spheroids (Figure 4a,b, respectively), treatment with IC_50_ and 2× IC_50_ of complex **1** significantly reduced the population of viable cells and concomitantly increased the population of dead cells. This is demonstrated by the reduction of average and normalized correlated total cell fluorescence (CTCF) of Calcein-AM, which is accompanied by the simultaneous increase in the CTCF of PI (Figure 4 bar graphs).

Furthermore, a closer inspection of the representative stained spheroids, in Figure 4, reveals that the cells that have been shed from the spheroid are indeed nonviable/dead, as they are stained red (Figure 4a,b). More intriguing, comparison of cells treated with the control (vehicle) to those treated with **1** reveals a significant reduction in the viable cells which are proliferating on the periphery of the spheroid (green fluorescence in the representative merged images in Figure 4) and a significant increase in unviable cells at the core of the spheroids (red fluorescence in the representative merged images in Figure 4) in a dose-dependent manner.

### 2.7 Mode of Cell Death Induced by **1** in RMS

**Figure 5:**
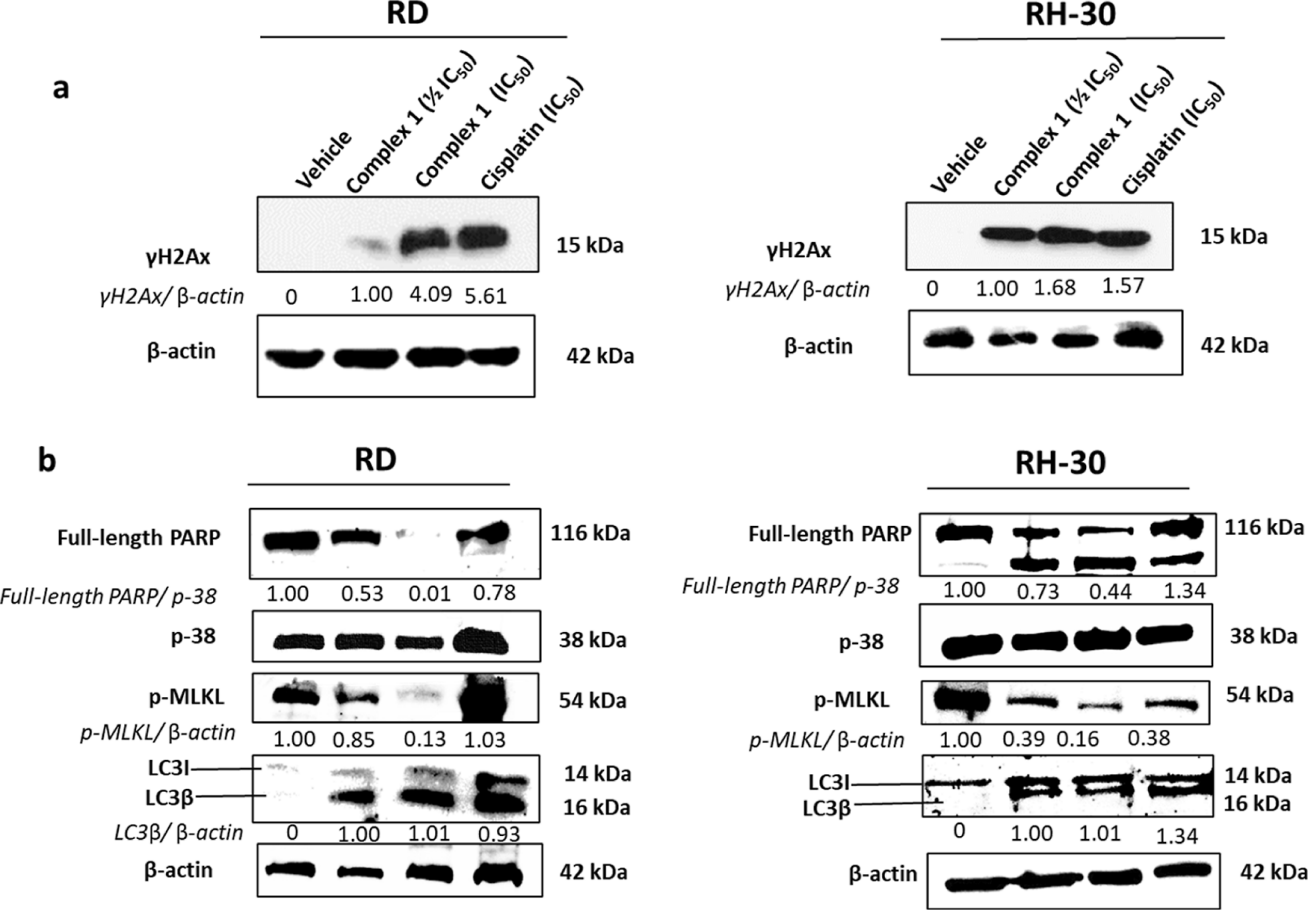
Western blot analyses of protein harvested from RMS cells treated with vehicle (0.1% DMSO), ^1^/_2_IC_50_ or IC_50_ of the complex **1**, or cisplatin for 48 h, and probed with antibodies indicating (a) DNA damage or (b) apoptosis, autophagy, or necroptosis. Either β-actin or p38 were used as a loading controls and densitometry readings were obtained using the ImageJ software. Protein expression levels are represented as a ratio of protein of interest/β-actin or p38 normalized to vehicle control samples (where possible). Blots are representative of two independent repeats.

Given that **1** shows potent cytotoxicity and ruthenium(II) arene complexes are generally known to induce DNA damage leading to cell death, the ability of **1** to induce DNA damage was studied by monitoring the levels of γH2AX, a protein related to the DNA damage pathway.^88−91^ The levels of γH2AX, a robust marker for DNA double-strand breaks, were markedly increased in a dose-dependent manner in both RMS cell lines (Figure 5a) indicating that **1** indeed induces DNA damage.

Cells treated with **1** were observed under a light microscope to see whether the DNA damage caused by the complex leads to cell death. The representative light microscopy images of the RD and RH-30 cells after treatment with **1** (Figure S26 in the Supporting Information) reveal that the cells display characteristic apoptotic morphology including membrane blebbing and cell shrinkage (red circles) and the presence of large vacuolar structures and vesicles (red arrows) associated with autophagy.^82,92,93^ In an effort to delineate the potential mechanism of cell death elicited by complex **1** in RMS cells, the effect on the protein levels of poly-ADP ribose polymerase (PARP), microtubule-associated protein light chain 3 (LC3β), and phosphorylated-mixed lineage kinase domain-like (*p*-MLKL) protein was investigated. These are involved in the three main forms of cell death: apoptosis, autophagy, and necroptosis.^94−97^ Generally, the total/full length PARP and *p*-MLKL protein levels are significantly reduced in RMS cells treated with **1** in a dose-dependent manner (Figure 5b), thus showing that the apoptotic pathway is activated. However, the reduction of *p*-MLKL levels suggests the inhibition of the necroptotic pathway. Western blotting also show an induction of autophagy, as the levels of LC3β increase in a dose-dependent manner in both RMS cell lines upon treatment with **1** (Figure 5b).

To further investigate the mechanism of action of **1** in RMS cells, the mechanism of apoptosis induction in RMS cells by **1** was explored, as there are two main apoptotic pathways: the intrinsic pathway and the extrinsic pathway. Of the two, the extrinsic pathway is the most ideal as it can also activate the intrinsic pathway, *via* the mitochondrial amplification loop, and is less prone to developed resistance by cancers.^98−100^ As a result, these two apoptotic pathways were investigated by studying the levels of cleaved caspase-8 and cleaved caspase-9 in RMS cells, which represent the extrinsic and intrinsic pathway, respectively. Furthermore, investigating the cleaved caspase-8 levels may further provide insight into the manner by which **1** suppresses necroptosis in RMS cells. This is due to caspase-8 being widely reported as a molecular switch for apoptosis and necroptosis, and increased levels of cleaved caspase-8 result in the repression of necroptosis.^101−103^

**Figure 6:**
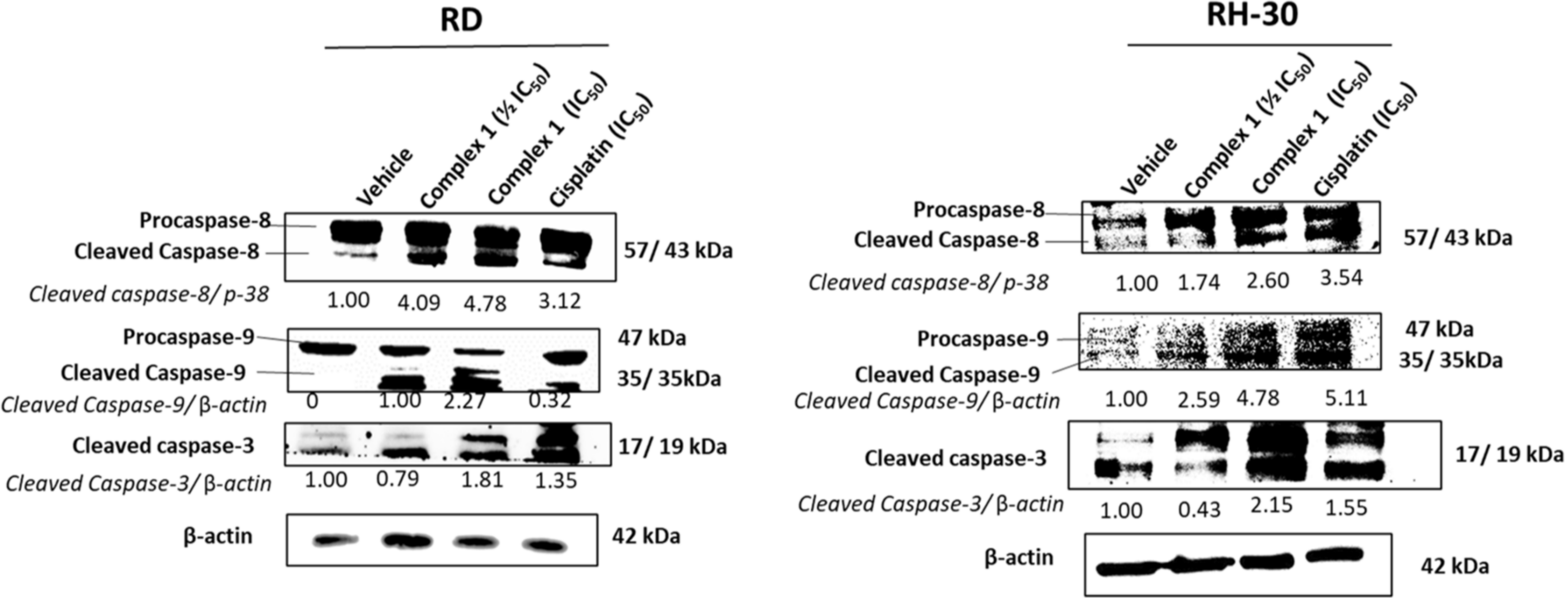
Western blot analyses of protein harvested from RMS cells treated with vehicle (0.1% DMSO), 1/2IC_50_ or IC_50_ of the complex **1**, or cisplatin for 48 h, and probed with antibodies indicating effector and executioner caspases of the apoptotic pathways. Either β-actin or p38 was used as a loading control, and densitometry readings were obtained using the ImageJ software. Protein expression levels are represented as a ratio of protein of interest/β-actin or p38 normalized to vehicle control samples (where possible). Blots are representative of two independent repeats.

The results from Western blot analysis, summarized in Figure 6, show that treatment of both cell lines with **1** results in increased levels of cleaved caspase-8 and caspase-9, which are the effectors of the extrinsic and intrinsic pathways, respectively. This correlates with increased levels of the active (cleaved) form of executioner caspase-3 (Figure 5) and its substrate PARP (Figure 4b). This unequivocally shows that **1** triggers both the intrinsic and extrinsic apoptosis pathways in RMS cells, and due to increasing levels of cleaved caspase-8, **1** results in the repression of necroptosis.

Overall, we have demonstrated the first example of a trinuclear organometallic complex that triggers both intrinsic and extrinsic apoptosis pathways in RMS cells.

## 3 Conclusions

In summary, we have developed a benzimidazole-based trinuclear ruthenium(II) arene complex that selectively targets RMS cancer cells. In particular, complex **1** containing the *p*-cymene ancillary ligand was generally the most active and the most selective, with cytotoxicity values comparable or superior to that of clinical drug, cisplatin, and selectivity indices significantly greater than that of cisplatin. In the long term cytotoxicity studies, we demonstrated that **1** not only shows enhanced cytotoxicity relative to clinically used cisplatin in RMS cells but is also less cytotoxic to nontumorigenic cells. Furthermore, we also confirmed that **1** was able to reduce the migration of the more aggressive and metastatic RH-30 aRMS cell line, suggesting that **1** may inhibit metastasis, which is one of the crucial hallmarks of cancer. In the 3D spheroid model, **1** exhibited potent cytotoxic activity in both RMS cell lines resulting in the overall reduction of spheroid size and the number of nonviable cells detached from the spheroid in a dose-dependent manner. This implies that **1** may reduce tumor size in *in vivo* animal studies. Finally, we established that **1** triggers DNA damage, initiates both the intrinsic and extrinsic apoptotic cell death pathways, and results in autophagy in both RMS cell lines, providing insight into the potential mechanism of action of the trinuclear ruthenium(II) complex.

Overall, this study demonstrates the first trinuclear ruthenium(II) complex **1** as a potentially effective chemotherapeutic agent for RMS, as **1** is the first of its kind to inhibit RMS cell survival (both short and long-term), proliferation (in 3D cultures), and migration. Importantly, **1** induces apoptosis and autophagy while being highly selective for cancer cells. On the basis of these findings, the therapeutic potential of trinuclear ruthenium(II) organometallic complexes offers promise for their future anticancer-metal-based drug development as RMS chemotherapeutic agents.

## 4 Experimental Procedures

### 4.1 Materials and Equipment

All reactions were conducted under an inert argon atmosphere unless specified otherwise. Reagents used for all reactions were purchased from commercial sources (Sigma-Aldrich, Combi-blocks) and used without additional purification. Solvents were reduced using a Büchi Rotavapor set to 40 °C. Reactions performed above room temperature were heated by using a hot plate and silicone oil. All aqueous solutions were prepared by using deionized water. Progression of all reactions was monitored by TLC using aluminum-backed Merck silica-gel F254 plates and were visualized under UV-lamp. Fulka Silica Gel 60, 40–63 μm was used to carry out all column chromatography.

All nuclear magnetic resonance spectra were recorded on either a Bruker X400 MHz spectrometer (^1^H at 399.95 MHz and ^13^C at 100.65 MHz) or a Varian Mercury XR300 MHz (^1^H at 299.95 MHz, ^13^C at 75.46 MHz) with tetramethylsilane (TMS) as the internal standard for chemical shifts. Chemical shifts and *J*-coupling values were reported in ppm and Hz, respectively. Infrared spectroscopy was conducted on a PerkinElmer Spectrum 100 FT-IR spectrometer using attenuated total reflectance (ATR) with bond vibrations measured in reciprocal centimeters (cm^–1^). Mass spectrometry (MS) determinations were carried out using electron impact (EI) on JEOL GCmatell instrument or Electrospray Ionization (ESI) on a Waters API Quattro Micro triple quadrupole mass spectrometer with data recorded using the positive mode. A Büchi Melting Point Apparatus B-540 machine was used to obtain the uncorrected melting points of each compound.

The purity of the complexes (**1–3**) was ascertained by high-performance liquid chromatography (HPLC) using an Agilent HPLC 1260 outfitted with an Agilent DAD 1260 UV/vis detector and an Agilent Pursuit 5 C18 column (5 μM, 150 mm × 4.6 mm). A mixture of solvent A (0.1% trifluoroacetic acid in deionized water) and solvent B (methanol) at a flow rate of 0.5 mL/min was used to elute the complexes. The gradient elution conditions were as follows: 90% solvent A between 0 and 2 min, 90–10% solvent A from 2 to 8 min, 10% solvent A from 8 to 20 min. All compounds are >95% pure by HPLC analysis.

The tris(2-(2-(pyridin-2-yl)-1*H*-benzo[*d*]imidazol-1-yl)ethyl)amine and tris(2-(2-(quinolin-2-yl)-1*H*-benzo[*d*]imidazol-1-yl)ethyl)amine precursors and the tris(2-(2-(quinolin-2-yl)-1*H*-benzo[*d*]imidazol-1-yl)ethyl)amine (**L1**) ligand were synthesized using methods reported in the literature.^46,104^

### 4.2 General Synthesis of the Trinuclear Ruthenium(II) Arene Complexes (**1–3**)

The tris(2-(2-(quinolin-2-yl)-1*H*-benzo[*d*]imidazol-1-yl)ethyl)amine (**L1**) ligand (1 equiv) was dissolved in a solution (5 mL) of EtOH and DCM (1:1) under argon. To this stirring solution, either [Ru(*p*-cymene)Cl_2_]_2_, [Ru(hexamethylbenzene)Cl_2_]_2_, or [Ru(benzene)Cl_2_]_2_ (1.5 equiv) was added, and the reaction mixture was stirred either at room temperature overnight (for **1** and **3**) or at 40 °C over 48 h (for **2**). Thereafter, a solution of ammonium hexafluorophosphate (3.1 equiv) in anhydrous ethanol (2 mL) was added, and the reaction mixture was stirred for an additional 30 min at room temperature. The contents of the reaction flask were subsequently filtered through Celite and rinsed with DCM (10 mL). Excess DCM was removed under reduced pressure, which resulted in a pale yellow (for **1** and **3**) or orange (for **2**) solid precipitate. The desired complexes were isolated by suction filtration and washed with cold ethanol (10 mL).

#### 4.2.1 Synthesis of the Trinuclear Ruthenium(II)-*p*-cymene Complex (**1**)

The tris(2-(2-(quinolin-2-yl)-1*H*-benzo[*d*]imidazol-1-yl)ethyl)amine ligand (0.0412 g, 0.0482 mmol) was reacted with [Ru(*p*-cymene)Cl_2_]_2_ (0.0443 g, 0.0723 mmol) at room temperature overnight. Thereafter, sodium hexafluorophosphate (0.0236 g, 0.145 mmol) was added to the reaction vessel, and the mixture was stirred at room temperature for an hour. The desired complex (**1**) was isolated as a pale-yellow solid (0.0956 g, 0.0460 mmol) by suction filtration. Yield: 95.5%. ^1^H NMR (300 MHz, DMSO) δ(ppm): 8.82 (d, *J* = 9.5 Hz, 3H), 8.32–7.93 (m, 11H), 7.94–7.38 (m, 16H), 6.30–6.03 (m, 9H), 5.89 (d, *J* = 5.4 Hz, 3H), 4.45 (d, *J* = 24.8 Hz, 6H), 2.86 (dd, *J* = 52.0, 44.4 Hz, 6H), 2.20 (d, *J* = 4.0 Hz, 9H), 2.08 (s, 3H), 0.84–0.65 (m, 9H), 0.66–0.45 (m, 9H). ^13^C NMR (151 MHz, DMSO) δ(ppm): 149.92, 147.97, 147.52, 141.00, 140.66, 136.22, 133.78, 130.32, 130.09, 129.03, 128.38, 127.14, 126.56, 119.45, 113.22, 107.04, 105.52, 103.62, 100.48, 86.82, 85.97, 85.14, 80.53, 30.63, 22.63, 21.96, 21.16, 18.78, 18.33. ^31^P NMR (162 MHz, DMSO) δ (ppm): −144.22 (hept, *J* = 711.4 Hz, PF_6_). FT-IR (ATR) ν (cm^–1^): 1592 (C=N_imine_), 836 (P–F). MP (°C): 249.1 (decomp.). MS (HR-ESI, *m*/*z*) observed: 574.7748 (100% [M-3PF_6_]^3+^) calcd, 574.7419.

#### 4.2.2 Synthesis of the Trinuclear Ruthenium(II)–Hexamethylbenzene Complex (**2**)

The tris(2-(2-(quinolin-2-yl)-1*H*-benzo[*d*]imidazol-1-yl)ethyl)amine ligand (0.0501 g, 0.0602 mmol) was reacted with [Ru(hexamethylbenzene)Cl_2_]_2_ (0.0608 g, 0.0903 mmol) at room temperature overnight. Thereafter, sodium hexafluorophosphate (0.0236 g, 0.145 mmol) was added to the reaction vessel and the mixture was stirred at room temperature for an hour. The desired complex (**2**) was isolated as a pale-yellow solid (0.113 g, 0.0592 mmol) via suction filtration. Yield: 98.3%. ^1^H NMR (300 MHz, DMSO) δ(ppm): 8.38 (dt, *J* = 28.0, 12.3 Hz, 5H), 8.12–7.78 (m, 11H), 7.75–7.28 (m, 16H), 4.23 (d, *J* = 63.1, 6H), 3.10–2.60 (m, 6H), 2.02 (s, *J* = 15.6 Hz, 4H), 1.76 (d, *J* = 6.0 Hz, 50H). ^13^C NMR (151 MHz, DMSO): δ 150.05, 149.44, 149.28, 148.34, 148.17, 148.01, 147.88, 146.45, 142.44, 140.48, 140.03, 139.51, 137.43, 136.70, 136.42, 133.10, 133.04, 130.35, 129.29, 128.96, 128.44, 128.26, 128.12, 126.77, 125.73, 124.06, 123.03, 121.66, 120.24, 119.84, 119.60, 118.83, 118.69, 113.41, 110.85, 99.47, 96.57, 95.09, 56.48, 52.06, 44.75, 43.93, 16.03, 15.71. ^31^P NMR (162 MHz, DMSO) δ (ppm): −144.19 (hept, *J* = 711.4 Hz, PF_6_). FT-IR (ATR) ν (cm^–1^): 1597 (C=N_imine_), 837 (P–F) MP (°C): 233.7 (decomp.). MS (HR-ESI, *m*/*z*) observed: 575.8063 (100% [M-3PF_6_]^3+^) calcd, 575.7959.

#### 4.2.3 Synthesis of the Trinuclear Ruthenium(II)–Benzene Complex (**3**)

The tris(2-(2-(quinolin-2-yl)-1*H*-benzo[*d*]imidazol-1-yl)ethyl)amine ligand (0.0503 g, 0.0605 mmol) was reacted with [Ru(benzene)Cl_2_]_2_ (0.0452 g, 0.0904 mmol) at 40 °C for 48 h. Thereafter, sodium hexafluorophosphate (0.0235 g, 0.145 mmol) was added to the reaction vessel, and the mixture was stirred at room temperature for an hour. The desired complex (**3**) was isolated as a pale-yellow solid (0.127 g, 0.0587 mmol) by suction filtration. Yield: 97.4%. ^1^H NMR (300 MHz, DMSO) δ (ppm): 9.00–8.72 (m, 3H), 8.65–8.43 (m, 2H), 8.33–7.41 (m, 26H), 6.14 (d, *J* = 9.2 Hz, 17H), 5.97 (s, 1H), 4.50 (d, *J* = 78.5 Hz, 5H), 2.90 (d, *J* = 43.7 Hz, 6H). ^13^C NMR (151 MHz, DMSO) δ (ppm): 149.57, 149.46, 148.77, 148.67, 147.78, 140.62, 140.51, 136.11, 135.95, 133.44, 130.16, 130.06, 129.97, 129.86, 129.23, 128.76, 128.41, 127.04, 126.24, 119.87, 119.25, 119.06, 113.06, 88.09, 86.48, 55.29, 52.35, 44.13. ^31^P NMR (162 MHz, DMSO) δ (ppm): −144.22 (hept, *J* = 711.4 Hz, PF_6_). FT-IR (ATR) ν (cm^–1^): 1595 (C=N_imine_), 833 (P–F). MP (°C): 229.8 (decomp.). MS (HR-ESI, *m*/*z*) observed: 491.7112 (100% [M-3PF_6_]^3+^) calcd, 491.6339.

### 4.3 Cell Culture

The estrogen-receptor-positive breast cancer cell line, MCF-7, and the cervical epidermoid carcinoma, Caski, were cultured in Roswell Park Memorial Institute (RPMI) 1640 medium (Sigma-Aldrich, USA). The triple-negative breast cancer cell line, MDA-MB-231, both rhabdomyosarcoma cell lines (RD and RH-30), the pancreatic ductal adenocarcinoma, PANC-1, and the human cervical cancer, HeLa, and the human skin fibroblast, FG-0, cell lines were cultured in Dulbecco’s modified Eagle medium (DMEM) (Sigma-Aldrich, USA). The metastatic pancreatic ductal adenocarcinoma, CFPAC-1, was cultured in Iscove’s modified Dulbecco’s medium. To enhance the culture system, both media were nourished with 10% heat-inactivated fetal bovine serum (FBS), 100 μg/mL streptomycin, and 100 U/mL penicillin. To sustain physiological pH and temperature, the cells were nurtured in a 5% CO_2_ environment, at 37 °C. Additionally, the culture media were replaced with fresh media every 48 to 72 h.

### 4.4 Cytotoxicity Studies

For the prescreen studies, cells were seeded at the required density and incubated under physiological conditions for 24 or 48 h to allow for adhesion. Thereafter, the cells were treated with either the vehicle control (0.1% DMSO), varying concentrations of the tested ruthenium(II) complexes, or cisplatin for 48 h. To quantify cell viability, the experiments were treated with the 3-(4,5-dimethylthiazol-2-yl)-2,5 diphenyltetrazolium bromide (MTT) salt according to the procedure described by Mosmann.^51^ The absorbance at 600 nm was measured using a GloMax Explorer Multimode Microplate Reader GM3500, Promega. At least three biological repeats in triplicate were performed from which the half maximal inhibitory concentration (IC_50_) was determined using GraphPad Prism v8.0.2 (GraphPad Software, California, USA). The selectivity index (S.I.) was determined by dividing the IC_50_ of the human skin fibroblast cell line (FG-0) by the IC_50_ of the cancer cell line.

### 4.5 Clonogenic Assay

Cells were seeded and treated at 60% confluency the following day with ^1^/_8_IC50, ^1^/_4_IC50, ^1^/_2_ IC_50_, IC_50_, and vehicle. After 24 h, 800–1500 cells were seeded in 35 mm dishes in drug-free medium. Formation of colonies was monitored, and the medium was changed when necessary. After 10–12 days, cells were fixed with 3:1 methanol: acetic acid and stained with crystal violet (Sigma-Aldrich, Missouri, USA). Dishes were imaged, and percentage colony area was determined using ImageJ v1.50i^105^ and the plugin ColonyArea.^106^ The colony area was determined for each compound concentration as a percentage of the vehicle-treated control.

### 4.6 Scratch Motility Assay

Cells were seeded in 6-well plates and allowed to adhere overnight to achieve 100% confluency. The following day, a sterile 200 μL pipet tip was used to make a vertical scratch in the cell monolayer of each well, and the cells were treated with 10 μM mitomycin C to inhibit proliferation and either the complex, cisplatin, or vehicle (0.1% DMSO). Cells were imaged at 0, 3, 6, 9, and 12 h postwound formation, and ImageJ v1.50i was used to calculate the area of the scratch.^105^ The total areas migrated was determined by subtracting the area for a specific time point from the area measured at 0 h.

### 4.7 Three-Dimensional Spheroid Studies

The rhabdomyosarcoma (RD and RH-30) cells were plated in a 96-well plate that was coated with 1.2% agarose (SeaKem LE Agarose 50004, Lonza, USA) to prevent cell adhesion. The cells were subsequently incubated under physiological conditions for 2 days to enable compact spheroid formation. Thereafter, the spheroids were treated with the ruthenium(II) complex of interest at 2× IC_50_, IC_50_, and ^1^/_2_IC_50_ concentrations, and the vehicle (0.1% DMSO) and cisplatin (at IC_50_) were included as controls. Images of the RMS spheroids were taken, using an EVOS M5000 Imaging System microscope, to monitor spheroid growth over 6 days. Three independent experiments with at least four replicates for each condition were performed. The area of the spheroids was measured using ImageJ v1.50i software.^105^

#### 4.7.1 Calcein-AM and PI Staining of Spheroids

On the sixth day, the RD and RMS spheroids were treated with three fluorescent stains to determine cell viability in the three-dimensional spheroids: calcein-AM (1 mg/mL, C1430, Invitrogen, USA), propidium iodide (PI) (1 mg/mL), and 4′,6-diamidino-2-phenylindole (DAPI) (500 μg/mL, Thermo Fisher Scientific, USA). The spheroids were subsequently incubated at 37 °C and 5% CO_2_ for an hour and imaged using an EVOS M5000 Imaging System microscope (Thermo Fisher Scientific, USA).

### 4.8 Western Blot Analyses

RMS cells were treated with either the vehicle control, **1,** or cisplatin for 48 h. Thereafter, the cells were lysed in whole-cell lysis buffer and Western blotting was carried out as described by Bleloch and co-workers.^82^ The primary antibodies used to incubate ECL membranes were rabbit polyclonal antibodies to phospho-histone H2AX (Ser139) (#2577), cleaved caspase-3 (Asp175) (#9661), PARP (#9542), caspase-9 (#9502), LC3β (#2775), rabbit monoclonal antibody to cleaved caspase-7 (Asp198) (D6H1) (#8438), and mouse monoclonal antibodies to caspase-8 (1C12) (#9746) from Cell Signaling Technology (Massachusetts, USA). Following primary antibody incubation, the membranes were incubated with goat antirabbit or goat antimouse horseradish peroxidase-conjugated secondary antibodies (Bio-Rad Laboratories, California, USA). Antibody reactive proteins were visualized using a WesternBright ECL HRP Substrate Kit (Advansta, California, USA). Either p38 or β-actin was used as loading controls, densitometry readings were obtained using ImageJ v1.50i,^105^ and protein expression levels were represented as the ratio of the protein of interest/p30 or β-actin loading controls normalized to the vehicle control sample. All Western blots are representative of at least two independent repeats.

## Supporting Information Available

NMR (^1^H, ^13^C, and ^31^P) and ESI-HRMS characterization, and the HPLC chromatograms of the novel complexes; UV/vis traces for the stability of the complexes **1–3** in DMSO and biological media; preliminary cytotoxic evaluations (at 10 and 20 μM) of the complexes **1–3** in breast (MCF-7 and MDA-MB-231), rhabdomyosarcoma (RD and RH-30), cervical (HeLa and CaSki), and pancreatic (PANC-1 and CFPAC-1) cancer cell lines; multidose screening of the complexes **1** and **2** in breast, rhabdomyosarcoma, and pancreatic cancer cell lines and a nontumorigenic cell line (FG-0); microscopic images of rhabdomyosarcoma cells after treatment and uncropped Western blots (PDF)
Molecular formula strings of complexes and biological data (CSV)

## Abbreviations used

3D: three-dimensional
aRMS: alveolar rhabdomyosarcoma
CTCF: correlated total cell fluorescence
DAPI: 4′,6-diamidino-2-phenylindole
eRMS: embryonal rhabdomyosarcoma
FDA: U.S. Food and Drug Administration
γH2Ax: phosphorylated histone H2AX
HER-2: human epidermal growth factor receptor 2
IC_50_: half-maximal inhibitory concentration
KRAS: Kristen rat sarcoma viral oncogene homologue
LC3β: microtubule-associated protein light chain 3
PARP: poly-ADP ribose polymerase
PGM: platinum-group metal
PI: propidium iodide
*p*-MLKL: phosphorylated-mixed lineage kinase domain-like protein
RMS: rhabdomyosarcoma
ROS-JNK: reactive oxygen species-Jun N-terminal kinase
VAC: vincristine, actinomycin D, and cyclophosphamide
