# Supplementary material for: Selective Targeting of Regulated Rhabdomyosarcoma Cells by Trinuclear Ruthenium(II)–Arene Complexes
Source: J Med Chem. 2024 Apr 3;67(8):6673–86. doi: 10.1021/acs.jmedchem.4c00256 (PMC11056987; doi:10.1021/acs.jmedchem.4c00256)
Supplement: Supplementary file 1 — jm4c00256_si_001.pdf [file jm4c00256_si_001.pdf]

Supporting Information

Selective Targeting of Regulated Rhabdomyosarcoma Cells by Trinuclear Ruthenium(II)-Arene Complexes

Athi Welsh, Karabo Serala, Sharon Prince and Gregory S. Smith*

a. Department of Chemistry, University of Cape Town, Rondebosch, 7700, South Africa

b. Department of Human Biology, University of Cape Town, Faculty of Health Science, Observatory, 7935, South Africa

*Corresponding Author: Professor Gregory S. Smith, Email: Gregory.Smith@uct.ac.za

Table of Contents

Figure S1: NMR Spectra of **1** in DMSO(d_6). 4
Figure S2: NMR Spectra of **2** in DMSO(d_6). 6
Figure S3: NMR Spectra of **3** in DMSO(d_6). 8
Figure S4: The HR-ESI Mass Spectrum of **1** recorded in the positive-ion mode (+ve). 8
Figure S5: The HR-ESI Mass Spectrum of **2** recorded in the positive-ion mode (+ve). 9
Figure S6: The HR-ESI Mass Spectrum of **3** recorded in the positive-ion mode (+ve). 9
Figure S7: The HPLC chromatogram of the complex **1**. 10
Figure S8: The HPLC chromatogram of the complex **2**. 11
Figure S9: The HPLC chromatogram of the complex **3**. 12
Figure S10: The UV/ Vis spectra of the complexes **1** – **3** in a 1% DMSO: 99% growth media (RPMI-1640) solution maintained at 37 °C. 13
Figure S11: The percentage cell survival of MCF-7 breast cancer cells 13
Figure S12: The percentage cell survival of MDA-MB-231 breast cancer cells. 14
Figure S13: The percentage cell survival of RD rhabdomyosarcoma cells. 14
Figure S14: The percentage cell survival of RH-30 rhabdomyosarcoma cells 15
Figure S15: The percentage cell survival of HeLa cervical cancer cells. 15
Figure S16: The percentage cell survival of Caski cervical cancer cells 16
Figure S17: The percentage cell survival of CFPAC-1 pancreatic cancer cells 16
Figure S18: The percentage cell survival of PANC-1 pancreatic cancer cells. 17

Figure S19: The percentage cell survival of MCF-7 breast cancer cells 17
Figure S20: The percentage cell survival of MDA-MB-231 breast cancer cells 18
Figure S21: The percentage cell survival of PANC-1 pancreatic cancer cells 18
Figure S22: The percentage cell survival of CFPAC-1 pancreatic cancer cells 19
Figure S23: The percentage cell survival of RD rhabdomyosarcoma cells. 19
Figure S24: The percentage cell survival of RH-30 rhabdomyosarcoma cells. 20
Figure S25: The percentage cell survival of FG-0 non-tumorigenic cells 20
Figure S26: Representative images (10X; EVOS M5000 imaging System) of the morphology of RD and RH-30 cells. 21
Figure S27: The uncropped Western blot in Figure 5a. 21
Figure S28: The uncropped Western blot in Figure 5b. 22
Figure S29: The uncropped Western blot in Figure 5b. 23
Figure S30: The uncropped Western blot in Figure 6. 24
Figure S31: The UV/ Vis spectra of the complexes **1** – **3** (at 10 µM) in 100% DMSO maintained at 37 °C. 25

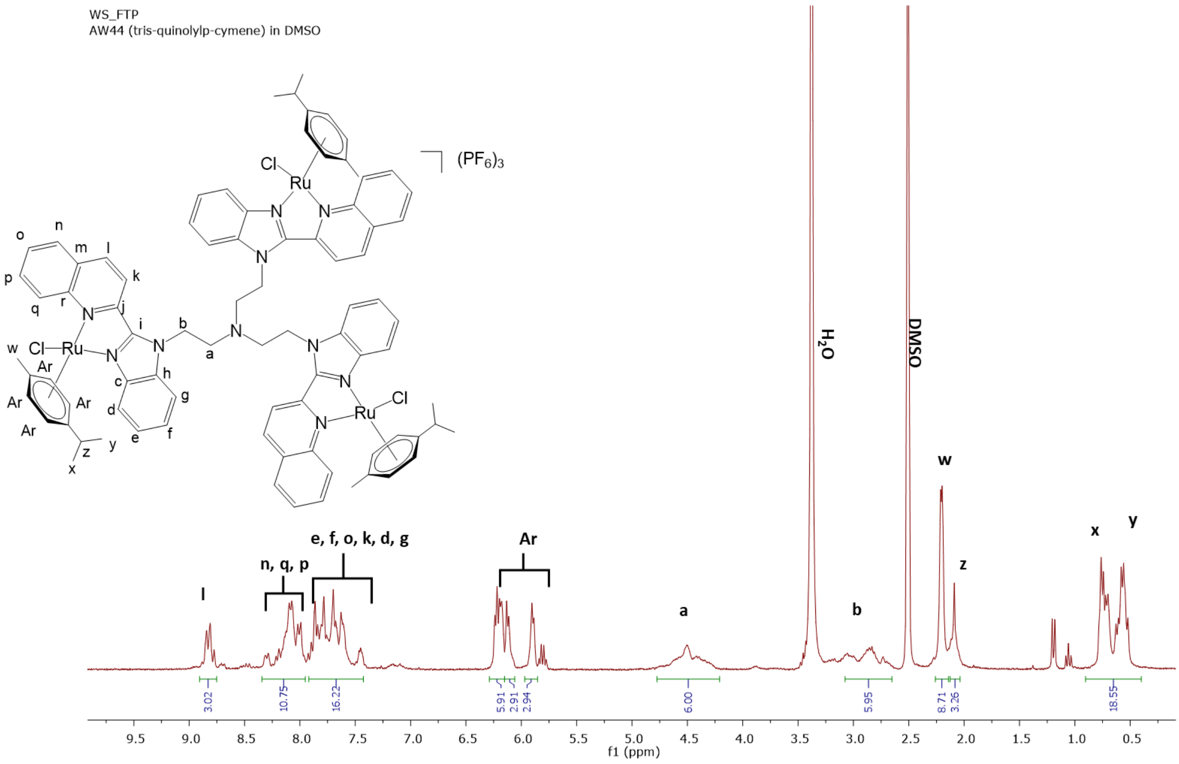
WS_FTP
AW44 (tris-quinolylp-cymene) in DMSO
(PF6)3
l
n, q, p
e, f, o, k, d, g
Ar
a
H2O
b
DMSO
w
z
x
y
3.02
10.75
16.22
5.91
2.91
2.94
6.00
5.95
8.71
3.26
18.55
f1 (ppm)

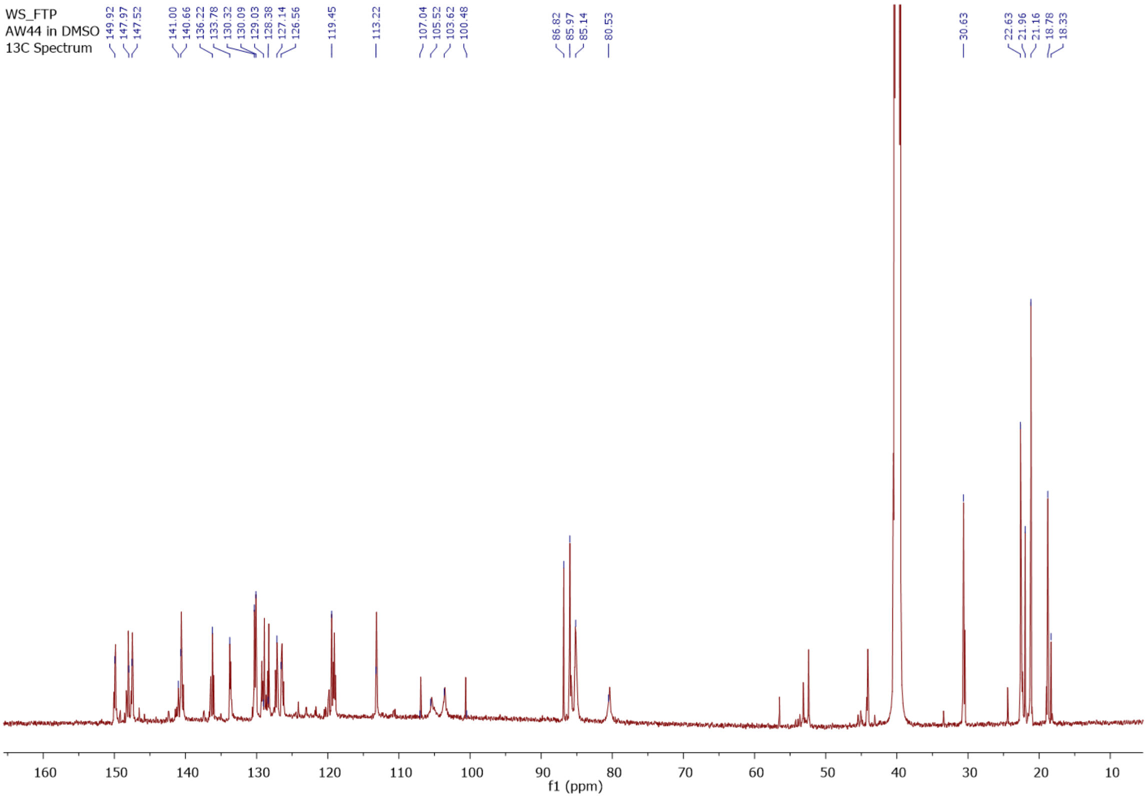
WS_FTP
AW44 in DMSO
13C Spectrum
149.92
147.97
147.52
141.00
140.66
136.22
133.78
130.32
130.09
129.03
128.38
127.14
126.56
119.45
113.22
107.04
105.52
103.62
100.48
86.82
85.97
85.14
80.53
30.63
22.63
21.96
21.16
18.78
18.33
f1 (ppm)

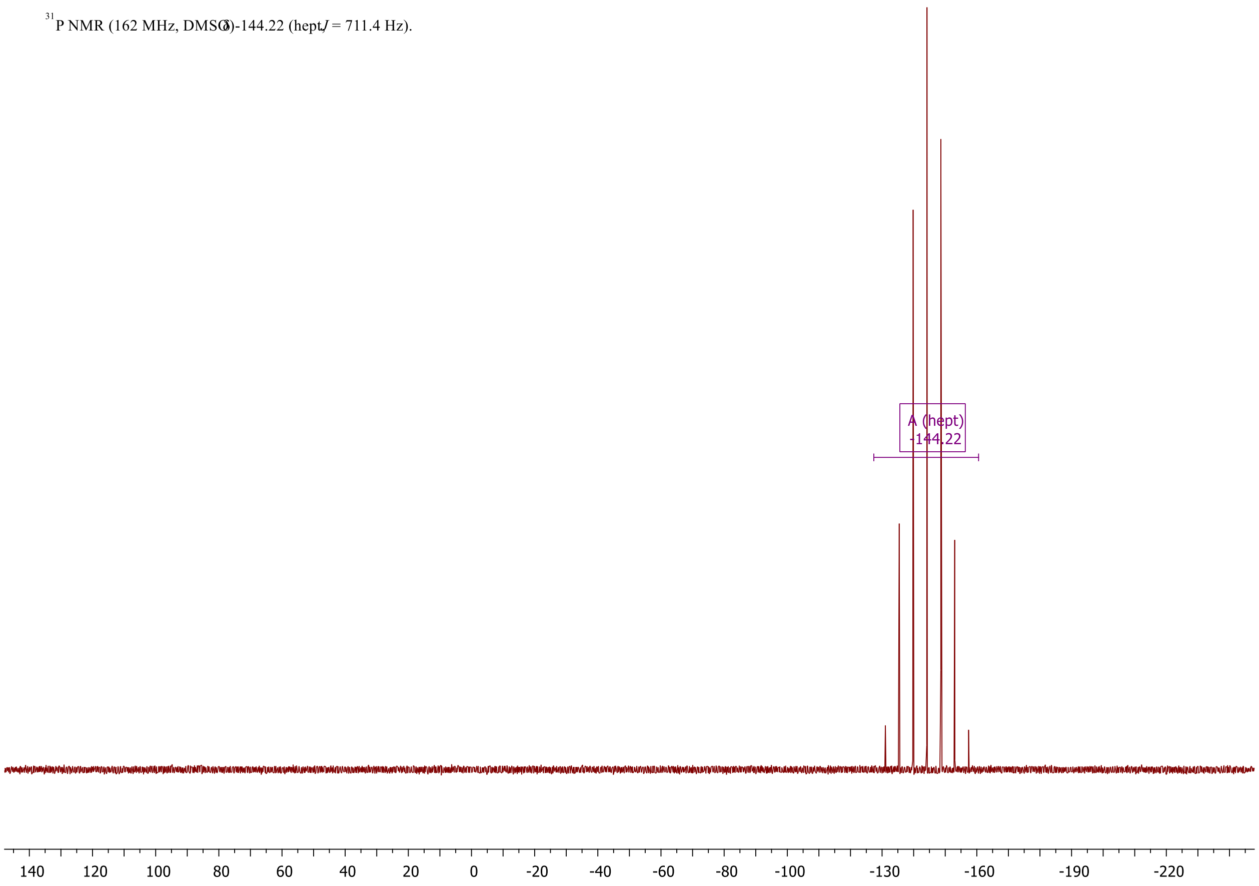

Figure S1: NMR Spectra of **1** in DMSO(d_6).

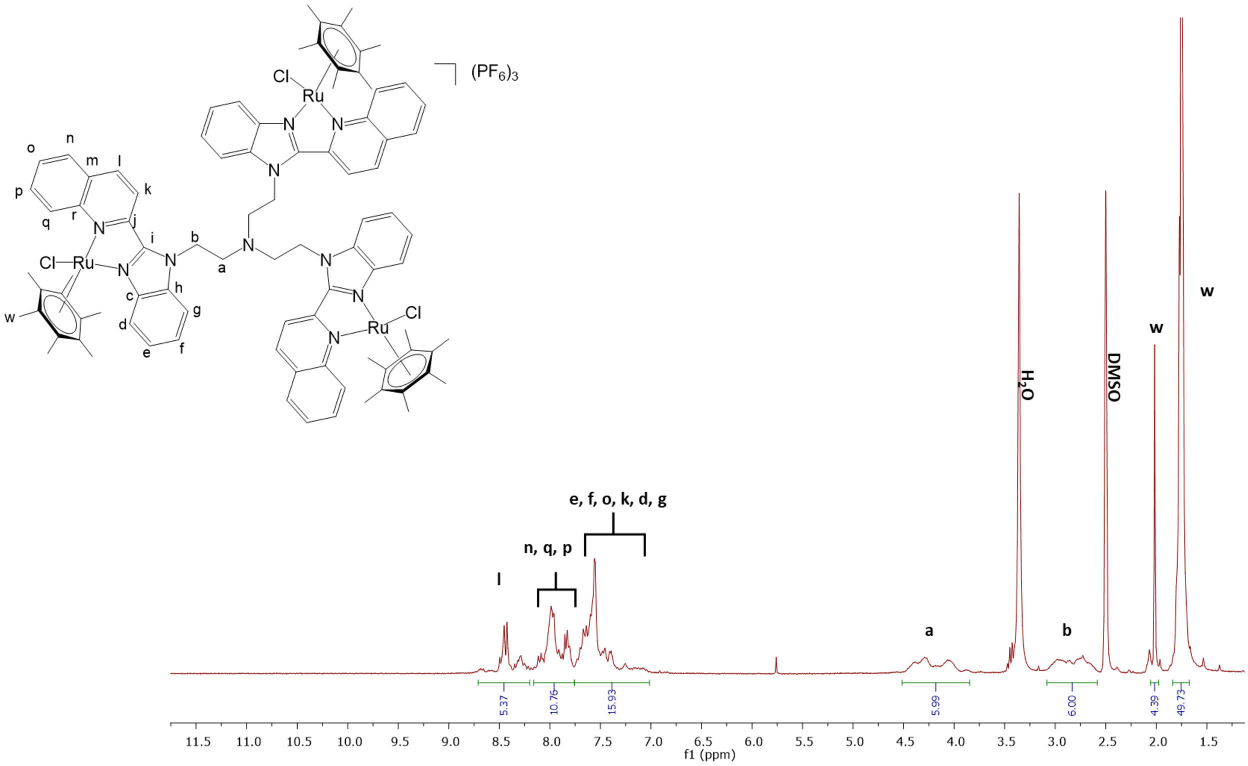
(PF6)3
Cl
Ru
N
a
b
c
d
e
f
g
h
i
j
k
l
m
n
o
p
q
r
w
l
n, q, p
e, f, o, k, d, g
a
b
H2O
DMSO
w
w
5.37
10.76
15.93
5.99
6.00
4.39
49.73
f1 (ppm)

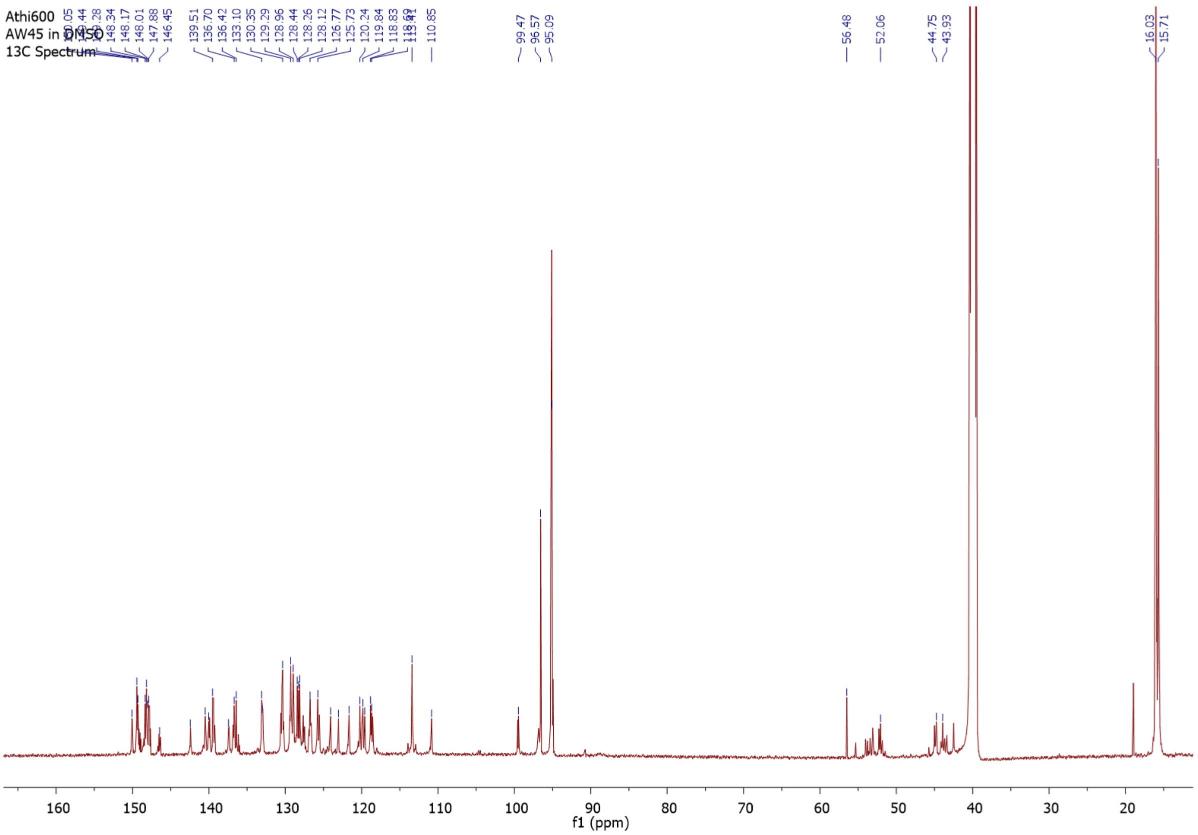
Athi600
AW45 in DMSO
13C Spectrum
148.34
148.17
148.01
147.88
146.45
139.51
136.70
136.42
133.10
130.35
129.29
128.96
128.44
128.26
128.12
126.77
125.73
120.24
119.84
118.83
110.85
99.47
96.57
95.09
56.48
52.06
44.75
43.93
16.03
15.71
f1 (ppm)

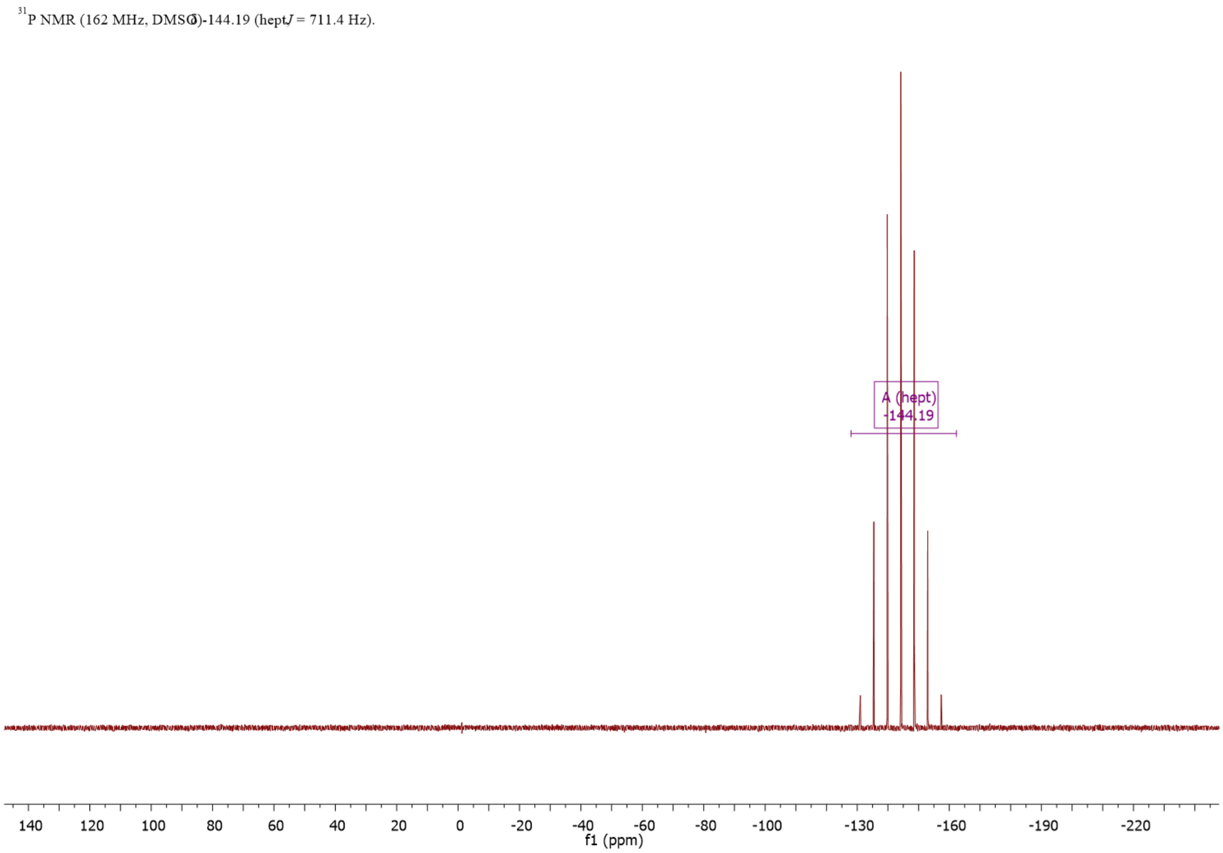

Figure S2: NMR Spectra of **2** in DMSO(d_6).

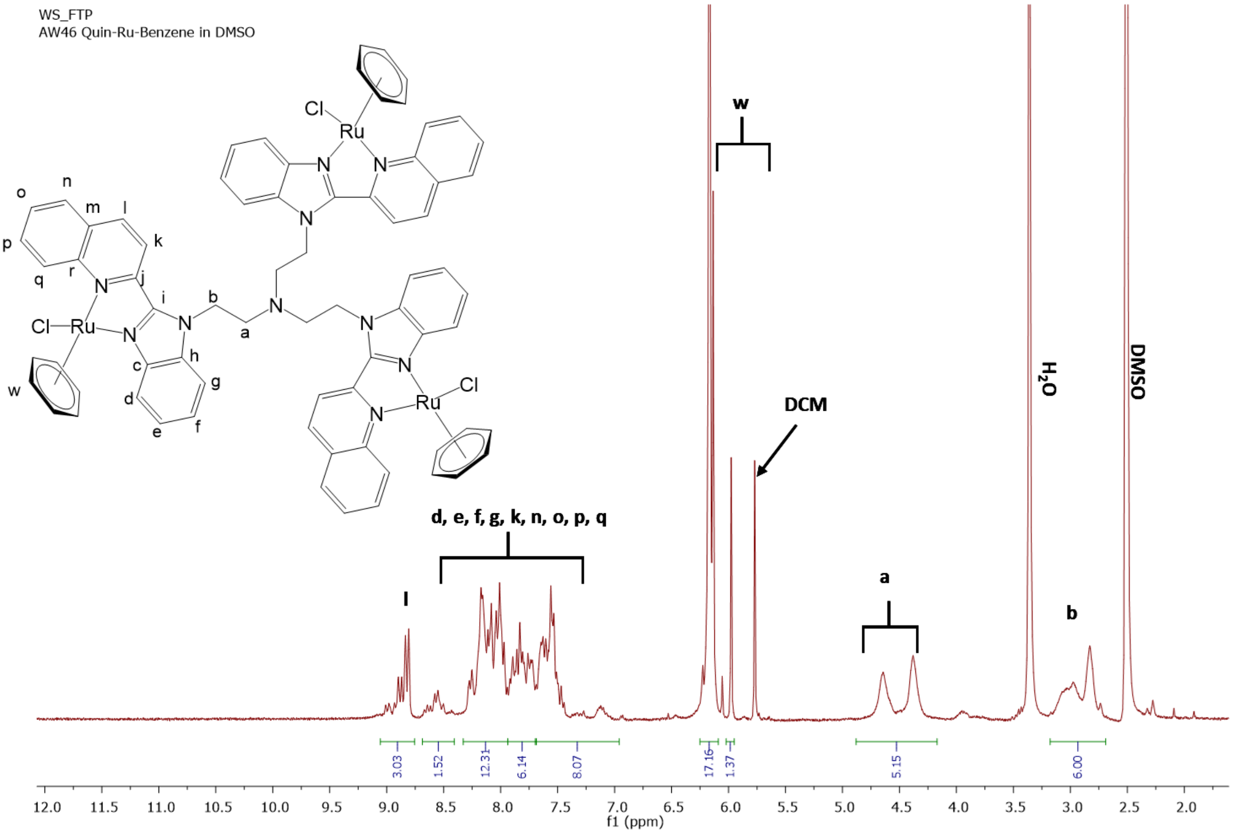
WS_FTP
AW46 Quin-Ru-Benzene in DMSO
w
DCM
H2O
DMSO
d, e, f, g, k, n, o, p, q
l
a
b
3.03
1.52
12.31
6.14
8.07
17.16
1.37
5.15
6.00
f1 (ppm)

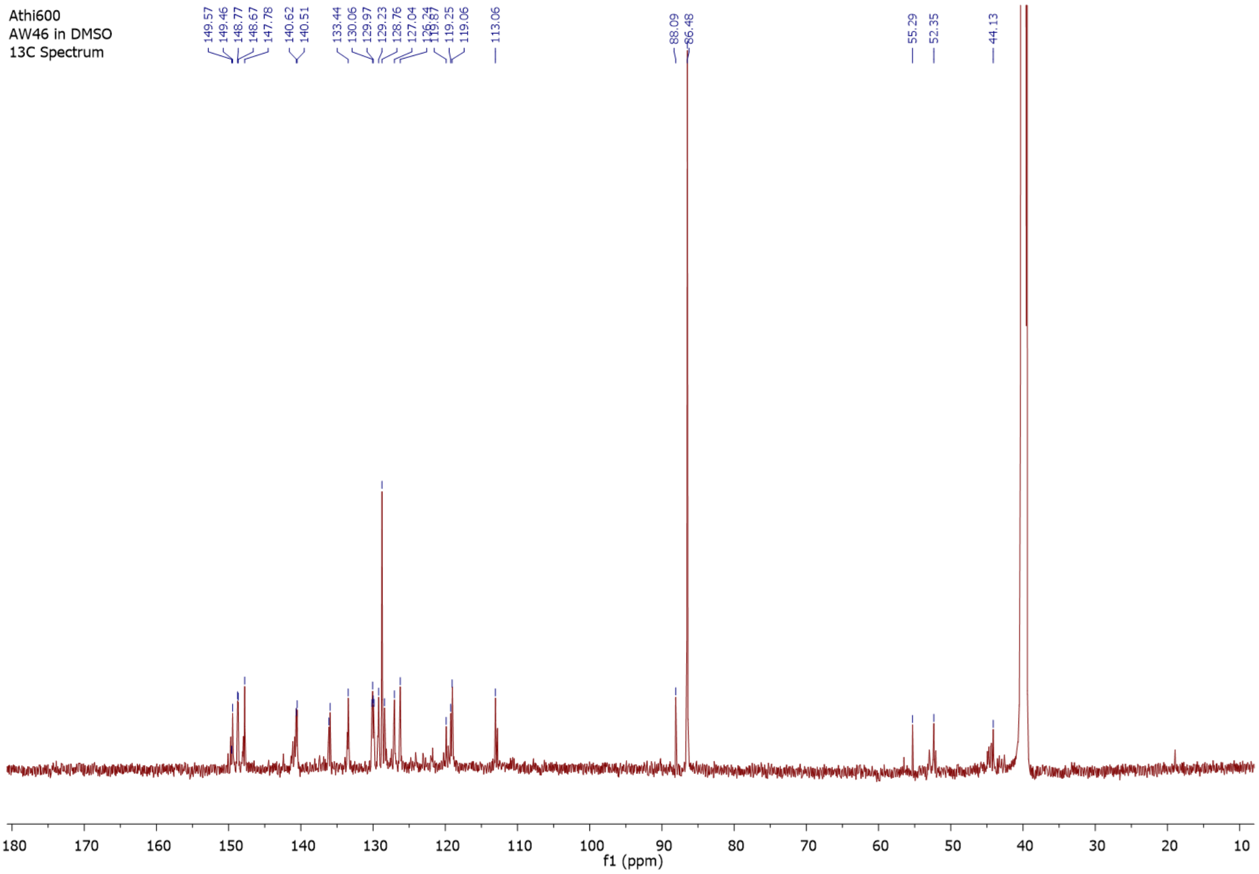
Athi600
AW46 in DMSO
13C Spectrum
149.57
149.46
148.77
148.67
147.78
140.62
140.51
133.44
130.06
129.97
129.23
128.76
127.04
126.24
119.87
119.25
119.06
113.06
88.09
86.48
55.29
52.35
44.13
f1 (ppm)

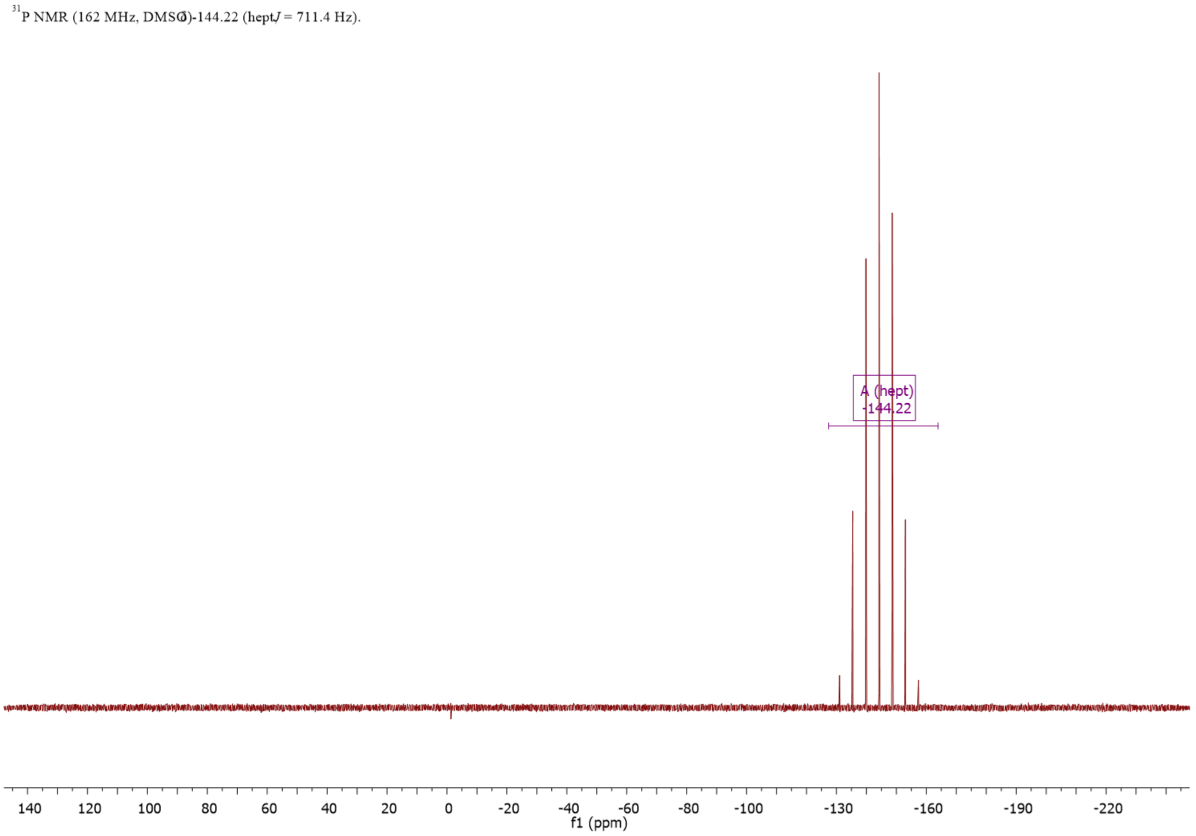

Figure S3: NMR Spectra of **3** in DMSO(d_6).

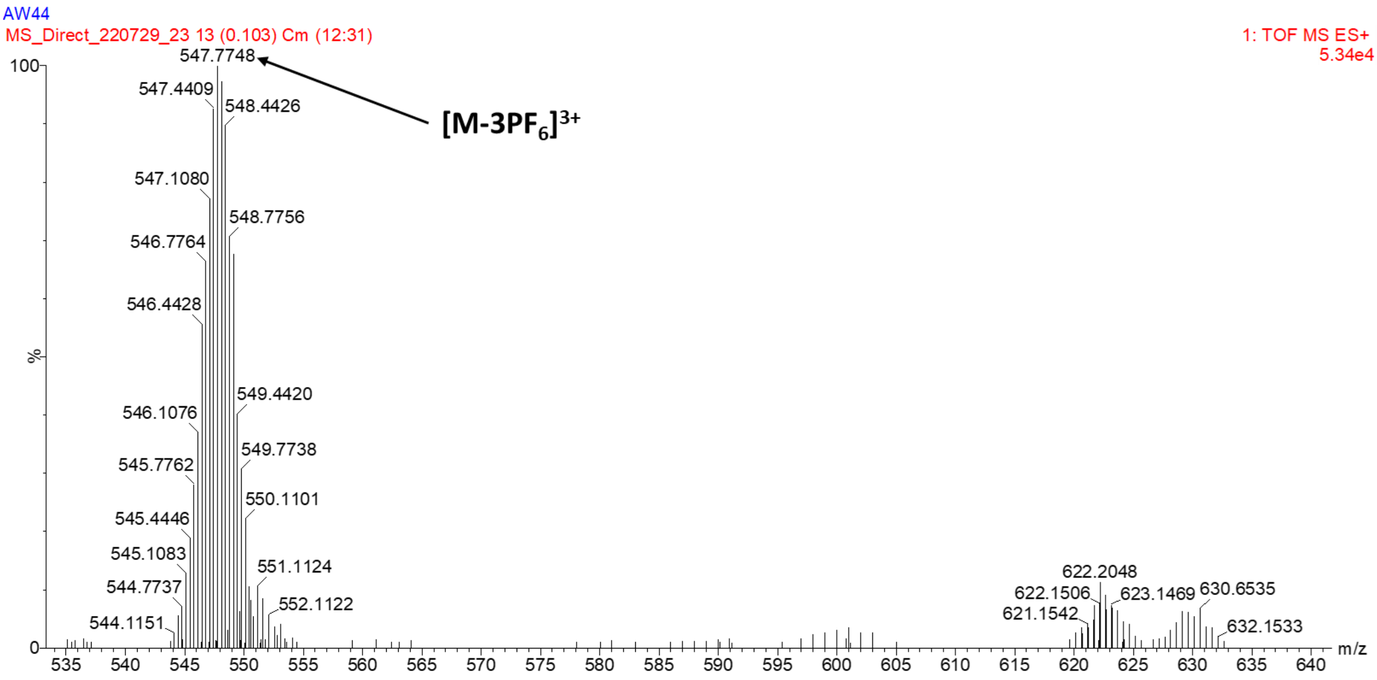

Figure S4: The HR-ESI Mass Spectrum of **1** recorded in the positive-ion mode (+ve), with the assigned base peak.

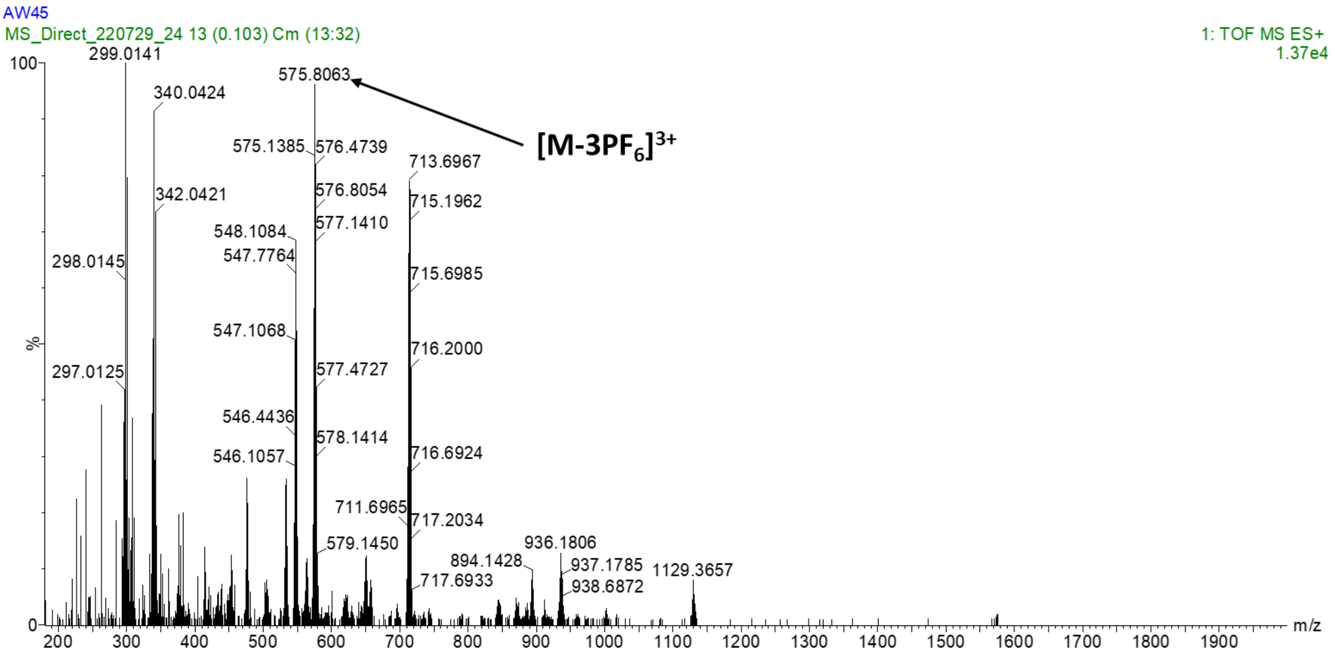

Figure S5: The HR-ESI Mass Spectrum of **2** recorded in the positive-ion mode (+ve), with the assigned base peak.

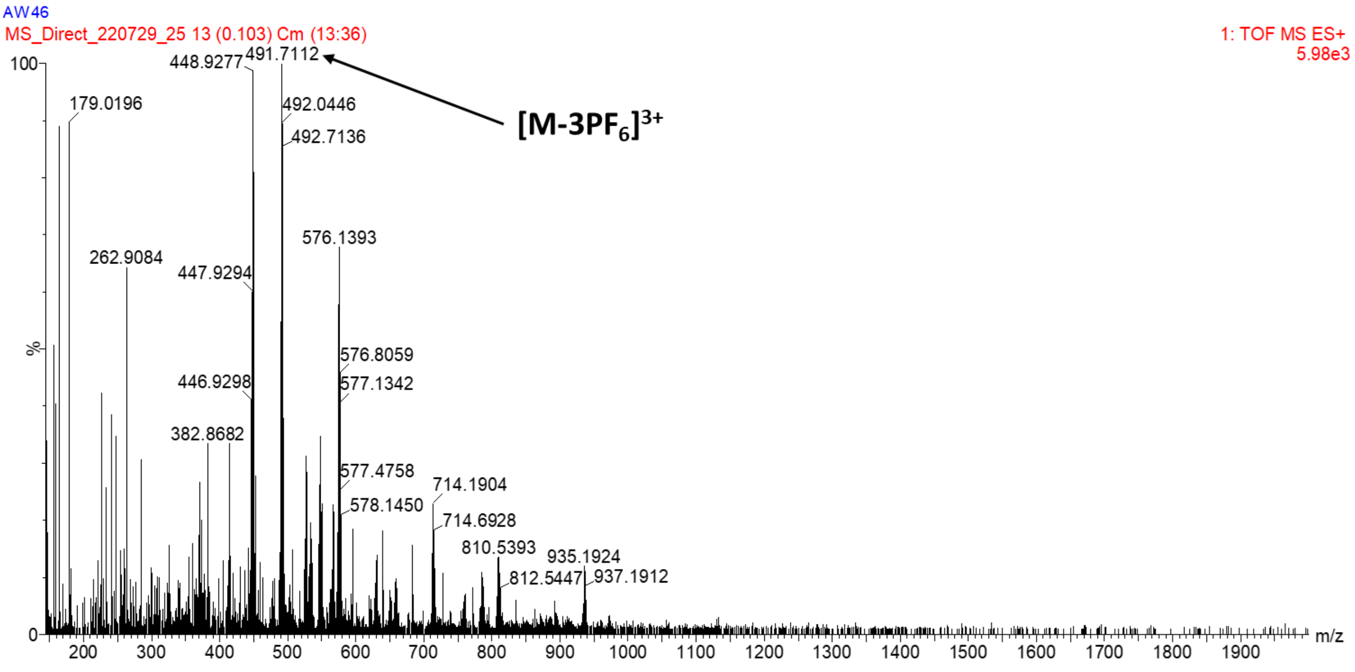

Figure S6: The HR-ESI Mass Spectrum of **3** recorded in the positive-ion mode (+ve), with the assigned base peak.

Single Injection Report

**Data file:**	2023-05-12 12-43-13+02-00-02.dx		
**Sequence Name:**	Arene Complexes	**Project Name:**	Petersen
**Sample name:**	AW 44	**Operator:**	SYSTEM
**Instrument:**	1260 HPLC	**Injection date:**	2023-05-12 12:44:09+02:00
**Inj. volume:**	30.000	**Location:**	P1-C2
**Acq. method:**	Standard HPLC Traces-0.1% TFA H2O-MeOH-1 ml.min.amx	**Type:**	Sample
**Processing method:**	GC_LC Area Percent_DefaultMethod.pmx	**Sample amount:**	0.00
**Manually modified:**	Manual Integration		

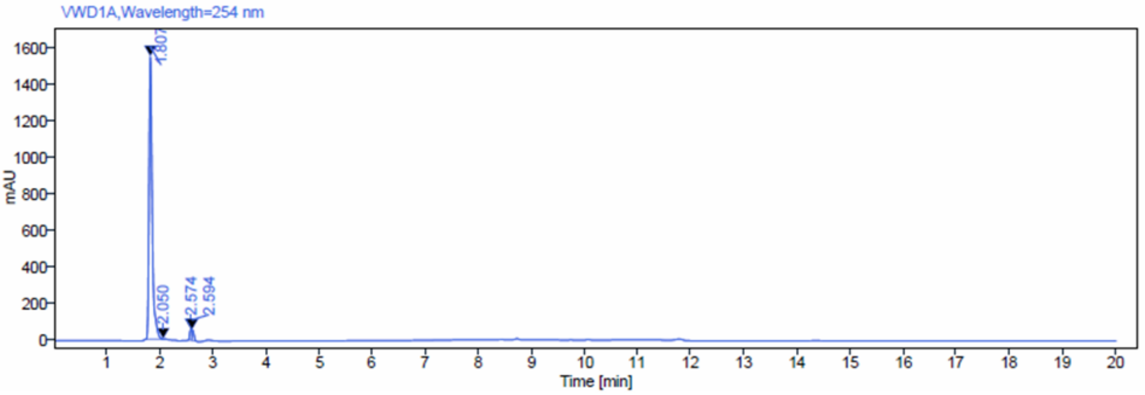

Signal: VWD1A,Wavelength=254 nm

RT [min]	Type	Width [min]	Area	Height	Area%	Name
1.807	MM m	0.06	6461.78	1552.27	95.66	
2.050	MM n	0.01	0.18	0.36	0.00	
2.574	MM m	0.04	139.23	58.08	2.06	
2.594	MM m	0.04	153.96	58.76	2.28	
		Sum	6755.16			

Figure S7: The HPLC chromatogram of the complex **1**.

Single Injection Report

**Data file:**	2023-05-12 13-04-12+02-00-03.dx		
**Sequence Name:**	Arene Complexes	**Project Name:**	Petersen
**Sample name:**	AW 45	**Operator:**	SYSTEM
**Instrument:**	1260 HPLC	**Injection date:**	2023-05-12 13:05:09+02:00
**Inj. volume:**	30.000	**Location:**	P1-C3
**Acq. method:**	Standard HPLC Traces-0.1% TFA H2O-MeOH-1 ml.min.amx	**Type:**	Sample
**Processing method:**	GC_LC Area Percent_DefaultMethod.pmx	**Sample amount:**	0.00
**Manually modified:**	Manual Integration		

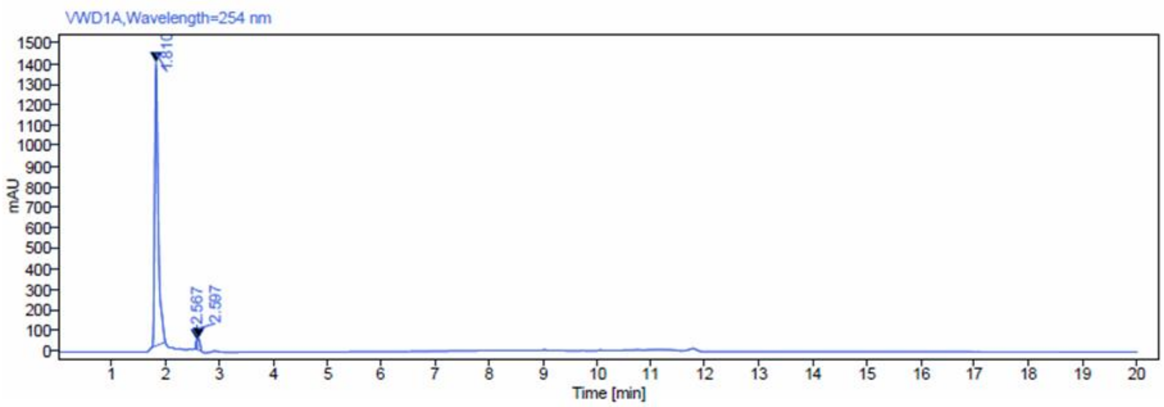

Signal: VWD1A,Wavelength=254 nm

RT [min]	Type	Width [min]	Area	Height	Area%	Name
1.810	MM m	0.07	6279.45	1376.32	96.17	
2.567	MM m	0.02	65.46	43.39	1.00	
2.597	MM m	0.05	184.52	55.11	2.83	
		Sum	6529.42			

Figure S8: The HPLC chromatogram of the complex **2**.

Single Injection Report

**Data file:**	2023-05-12 13-25-12+02-00-04.dx		
**Sequence Name:**	Arene Complexes	**Project Name:**	Petersen
**Sample name:**	AW 46	**Operator:**	SYSTEM
**Instrument:**	1260 HPLC	**Injection date:**	2023-05-12 13:26:10+02:00
**Inj. volume:**	30.000	**Location:**	P1-C4
**Acq. method:**	Standard HPLC Traces-0.1% TFA H2O-MeOH-1 ml.min.amx	**Type:**	Sample
**Processing method:**	GC_LC Area Percent_DefaultMethod.pmx	**Sample amount:**	0.00
**Manually modified:**	Manual Integration		

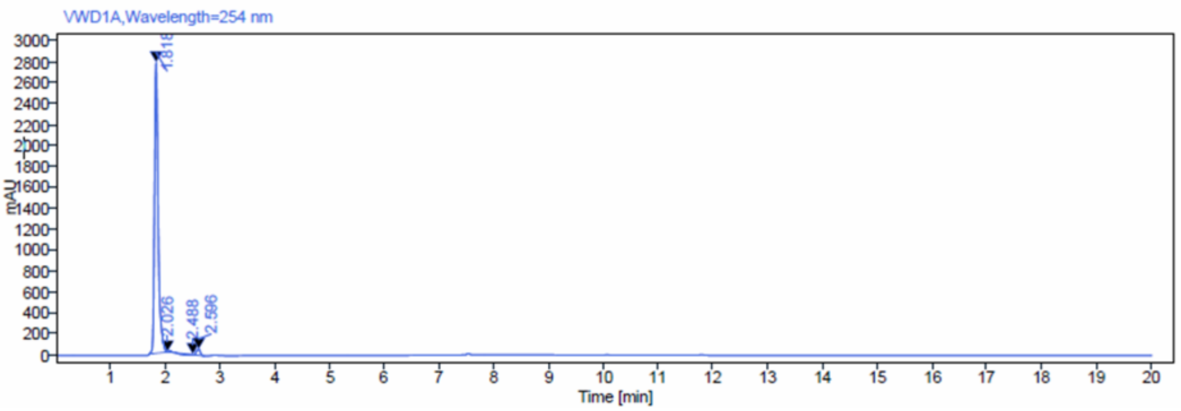

Signal: VWD1A,Wavelength=254 nm

RT [min]	Type	Width [min]	Area	Height	Area%	Name
1.818	MM m	0.07	12824.24	2776.64	97.22	
2.026	MM n	0.01	1.36	2.25	0.01	
2.488	MM n	0.40	96.65	2.92	0.73	
2.596	MM m	0.07	269.09	58.85	2.04	
		Sum	13191.34			

Figure S9: The HPLC chromatogram of the complex **3**.

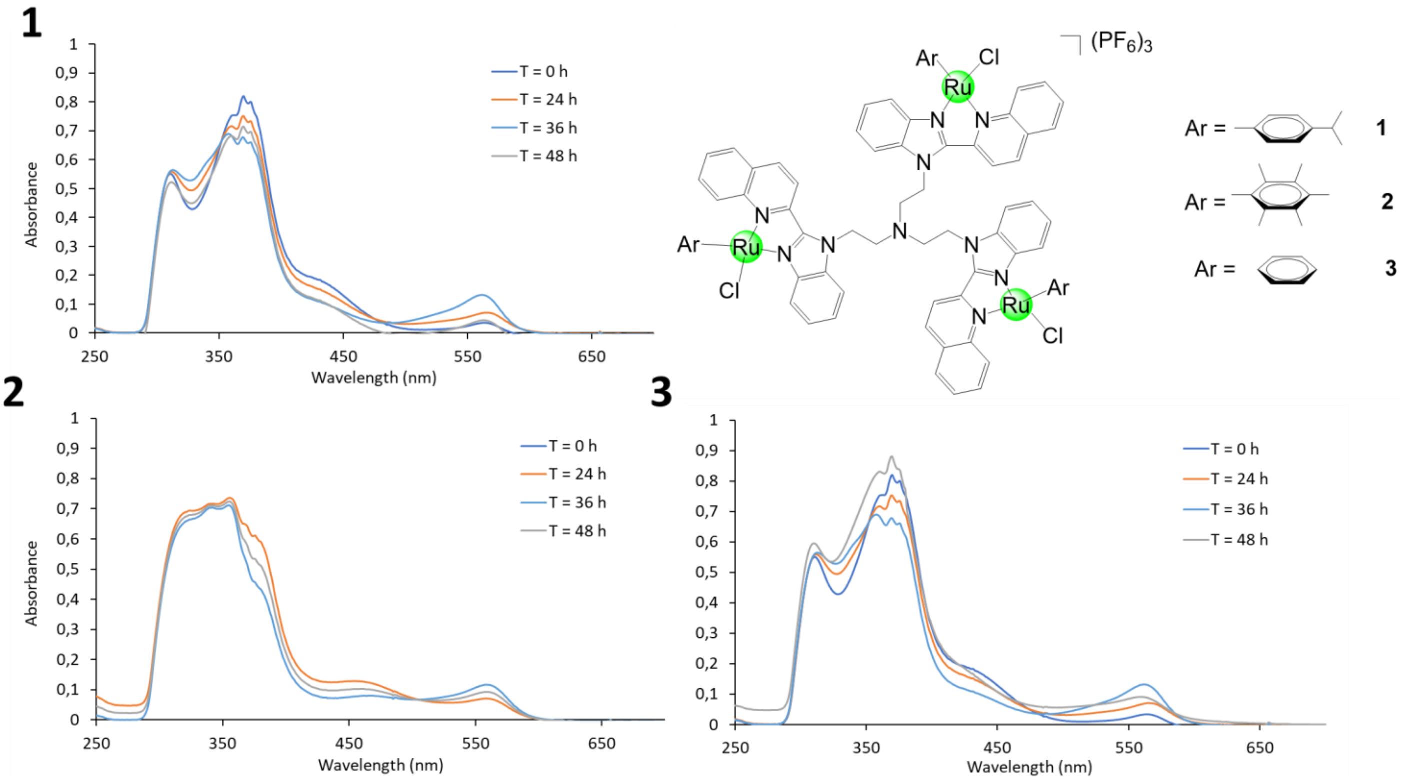

Figure S10: The UV/ Vis spectra of the complexes **1** – **3** (at concentrations ranging between 8 and 10 μM) in a 1% DMSO: 99% growth media (RPMI-1640) solution maintained at 37 °C.

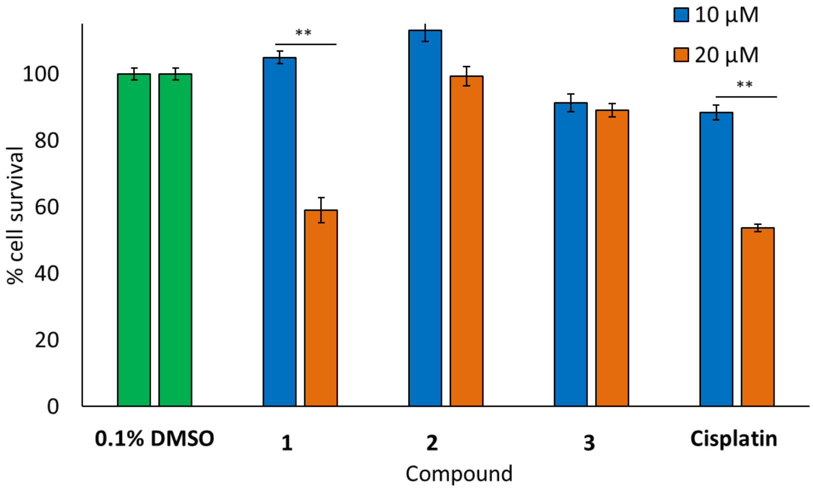

	% cell viability	
**Compound**	10 μM	20 μM
**1**	104.9	59.1
**2**	113.1	99.4
**3**	91.3	89.1
**Cisplatin**	88.4	53.5

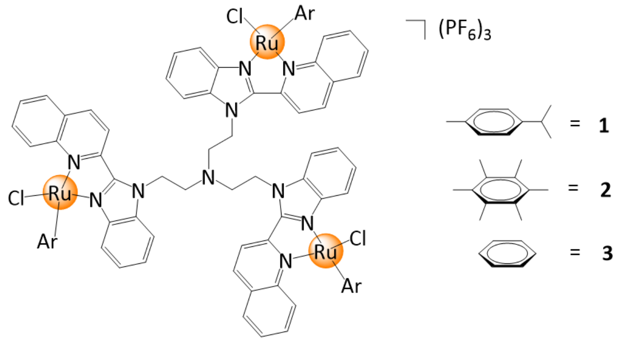

Figure S11: The percentage cell survival of MCF-7 breast cancer cells as determined by MTT assays. The cancer cells were treated with either 0.1% DMSO (vehicle control), the test compounds (**1** – **3**), or cisplatin at 10 μM or 20 μM. * = $p \leq 0.05$, thus statistically significant and ** = $p \leq 0.01$, thus very statistically significant

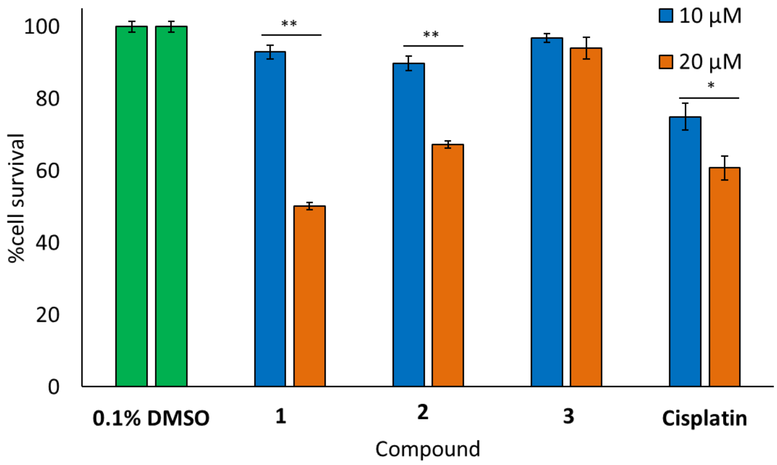

	% cell viability	
**Compound**	10 µM	20 µM
**1**	92.9	50.2
**2**	89.8	67.3
**3**	96.8	93.9
**Cisplatin**	74.1	60.7

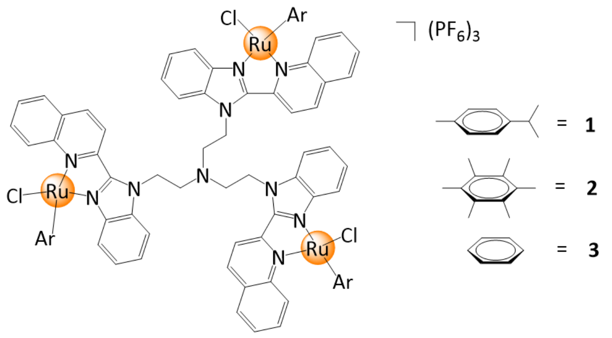

Figure S12: The percentage cell survival of MDA-MB-231 breast cancer cells as determined by MTT assays. The cancer cells were treated with either 0.1% DMSO (vehicle control), the test compounds (**1** – **3**), or cisplatin at 10 µM or 20 µM. * = $p \leq 0.05$, thus statistically significant and ** = $p \leq 0.01$, thus very statistically significant.

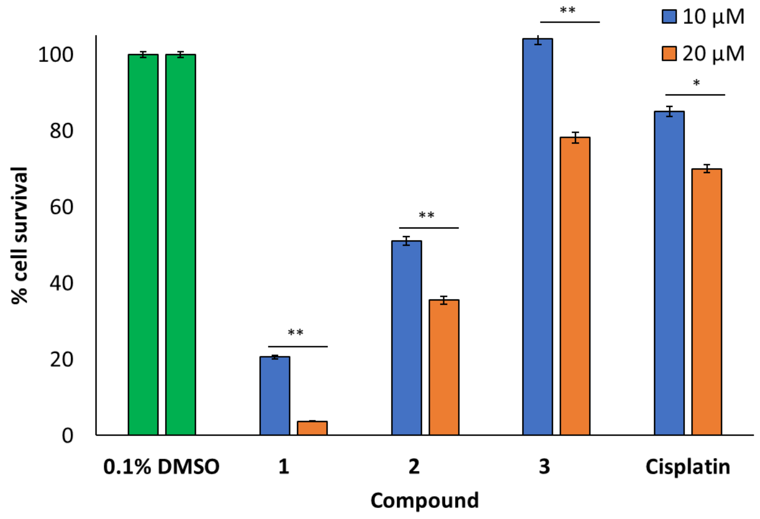

	% cell viability	
**Compound**	10 µM	20 µM
**1**	20.6	3.8
**2**	51.1	35.5
**3**	104.2	78.2
**Cisplatin**	85.1	70

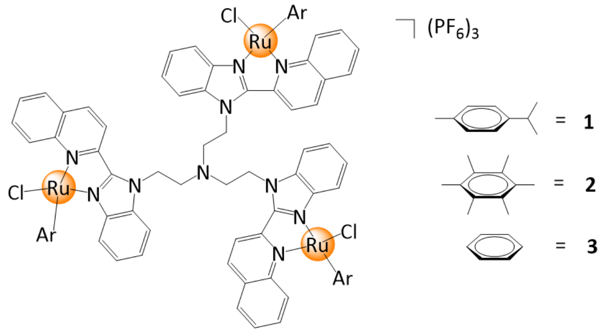

Figure S13: The percentage cell survival of RD rhabdomyosarcoma cells as determined by MTT assays. The cancer cells were treated with either 0.1% DMSO (vehicle control), the test compounds (**1** – **3**), or cisplatin at 10 µM or 20 µM. * = $p \leq 0.05$, thus statistically significant and ** = $p \leq 0.01$, thus very statistically significant.

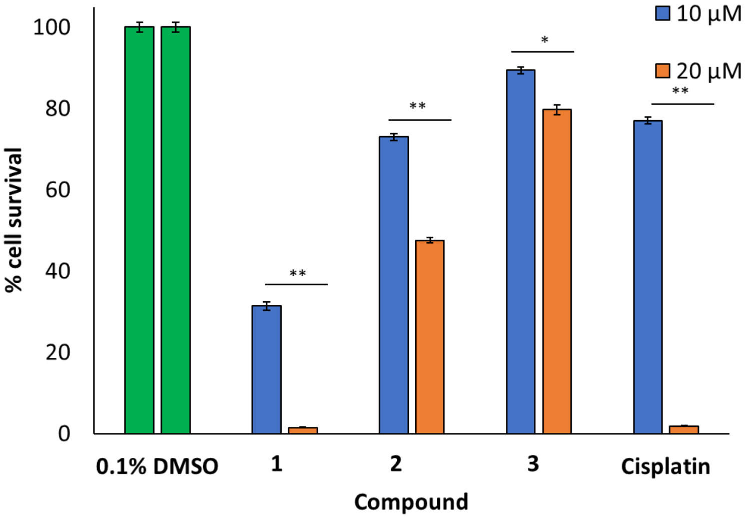

	% cell viability	
**Compound**	10 µM	20 µM
**1**	31.4	1.5
**2**	73.0	47.5
**3**	89.4	79.7
**Cisplatin**	77.0	1.9

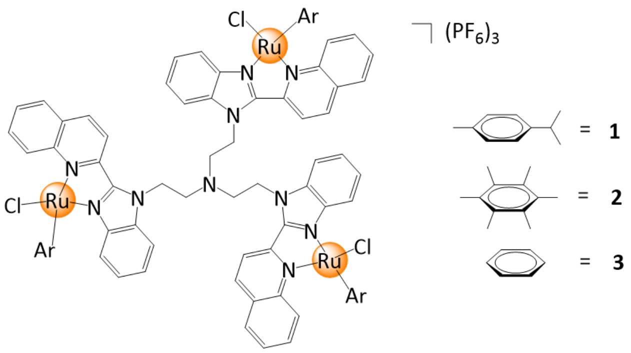

Figure S14: The percentage cell survival of RH-30 rhabdomyosarcoma cells as determined by MTT assays. The cancer cells were treated with either 0.1% DMSO (vehicle control), the test compounds (**1** – **3**), or cisplatin at 10 µM or 20 µM. * = $p \leq 0.05$, thus statistically significant and ** = $p \leq 0.01$, thus very statistically significant.

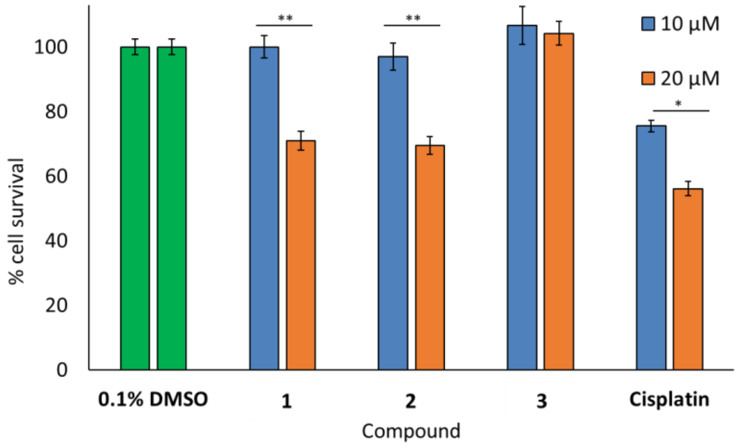

	% cell viability	
**Compound**	10 µM	20 µM
**1**	100.1	70.9
**2**	96.9	69.4
**3**	106.7	104.1
**Cisplatin**	75.4	56.8

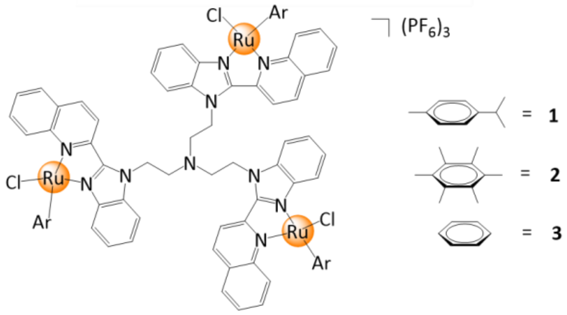

Figure S15: The percentage cell survival of HeLa cervical cancer cells as determined by MTT assays. The cancer cells were treated with either 0.1% DMSO (vehicle control), the test compounds (**1** – **3**), or cisplatin at 10 µM or 20 µM. * = $p \leq 0.05$, thus statistically significant and ** = $p \leq 0.01$, thus very statistically significant.

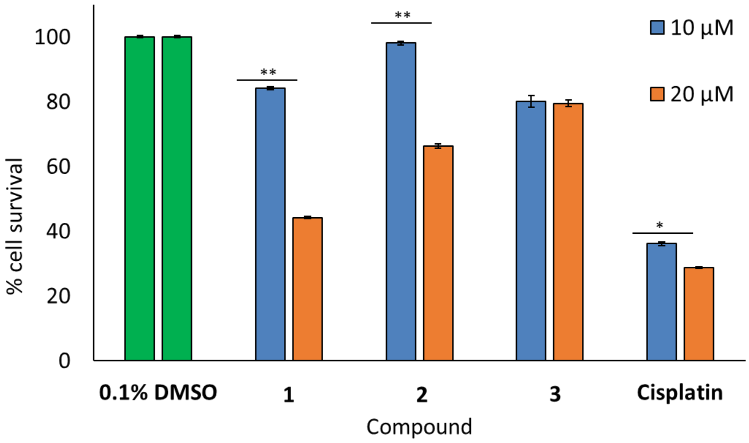

Compound	% cell viability 10 µM	20 µM
**1**	84.2	44.2
**2**	98.1	66.3
**3**	80.1	79.5
**Cisplatin**	36.2	28.8

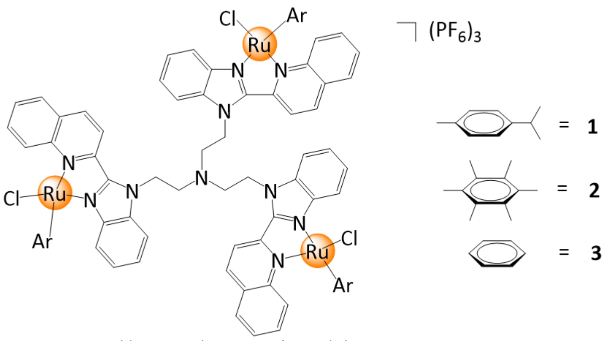

Figure S16: The percentage cell survival of Caski cervical cancer cells as determined by MTT assays. The cancer cells were treated with either 0.1% DMSO (vehicle control), the test compounds (**1** – **3**), or cisplatin at 10 µM or 20 µM. * = $p \leq 0.05$, thus statistically significant and ** = $p \leq 0.01$, thus very statistically significant.

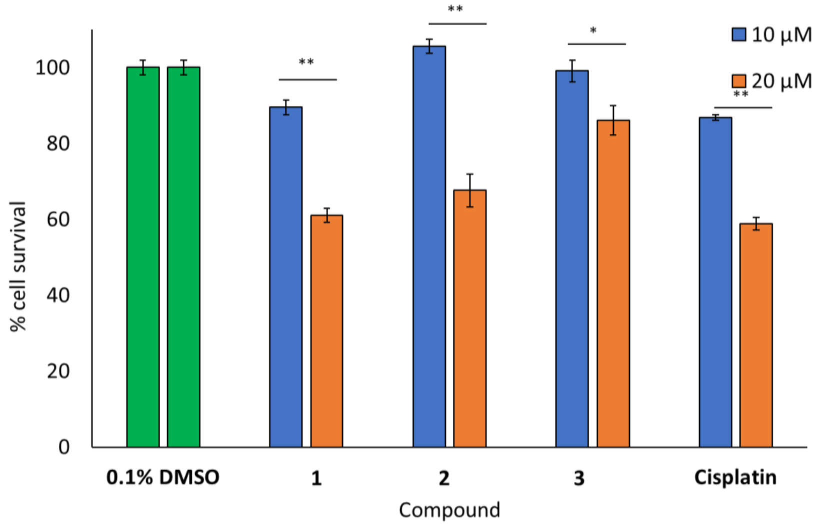

Compound	% cell viability 10 µM	20 µM
**1**	89.5	61.1
**2**	105.6	67.6
**3**	99.1	86.0
**Cisplatin**	86.9	58.8

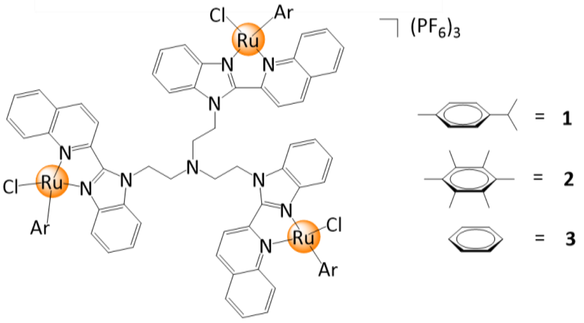

Figure S17: The percentage cell survival of CFPAC-1 pancreatic cancer cells as determined by MTT assays. The cancer cells were treated with either 0.1% DMSO (vehicle control), the test compounds (**1** – **3**), or cisplatin at 10 µM or 20 µM. * = $p \leq 0.05$, thus statistically significant and ** = $p \leq 0.01$, thus very statistically significant.

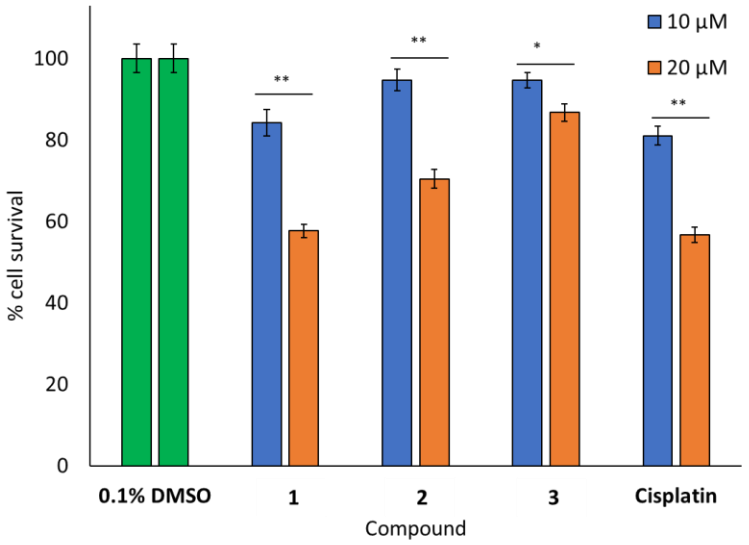

	% cell viability	
Compound	10 µM	20 µM
**1**	84.2	57.6
**2**	94.7	70.4
**3**	94.7	86.7
**Cisplatin**	81.0	56.6

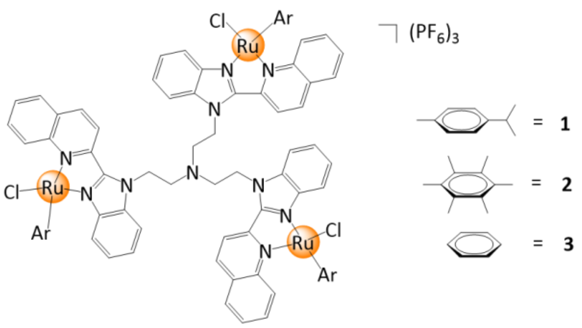

Figure S18: The percentage cell survival of PANC-1 pancreatic cancer cells as determined by MTT assays. The cancer cells were treated with either 0.1% DMSO (vehicle control), the test compounds (**1** – **3**), or cisplatin at 10 µM or 20 µM. * = $p \leq 0.05$, thus statistically significant and ** = $p \leq 0.01$, thus very statistically significant.

Compound	IC_{50} (µM)
**1**	23.54 ± 1.48
**2**	32.92 ± 1.05
**Cisplatin**	24.92 ± 1.03

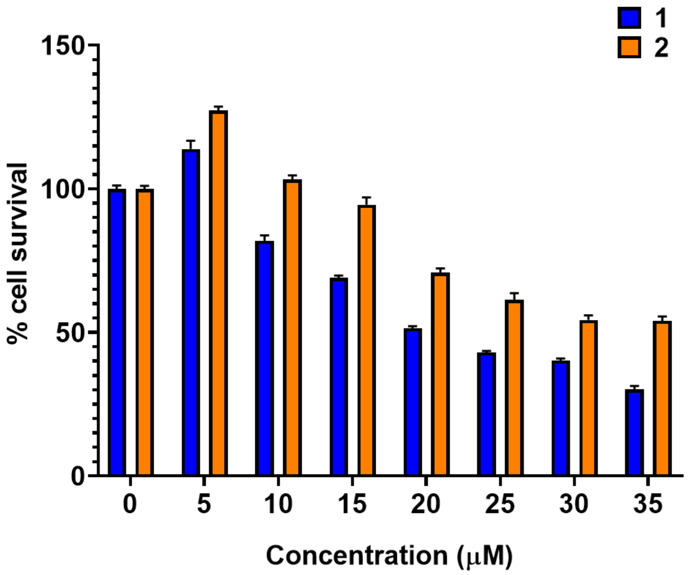

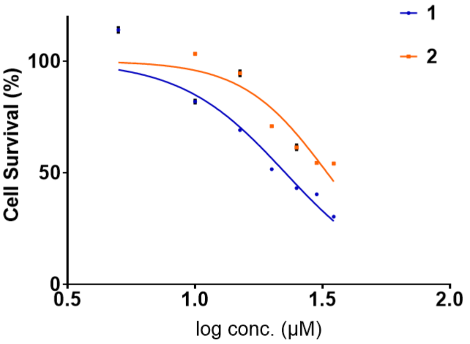

Figure S19: The percentage cell survival of MCF-7 breast cancer cells as determined by MTT assays. The cancer cells were treated with either 0.1% DMSO (vehicle control), the test compounds (**1** and **2**) at concentrations between 5 µM and 35 µM. * = $p \leq 0.05$, thus statistically significant and ** = $p \leq 0.01$, thus very statistically significant.

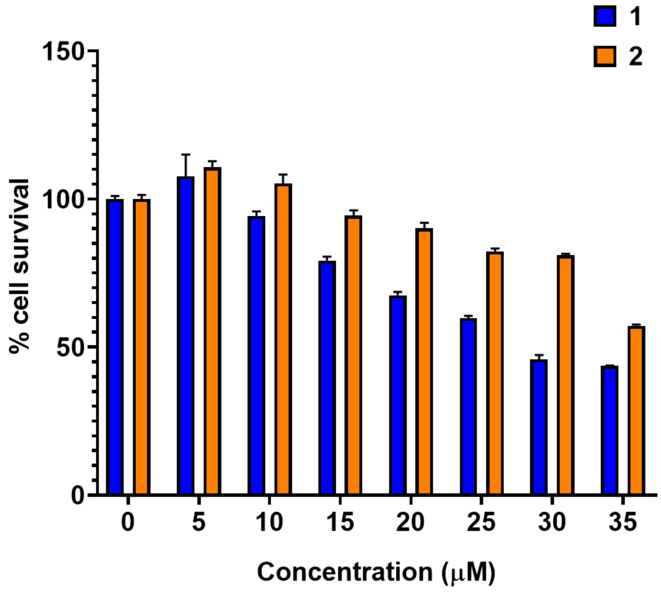

Compound	IC$_{50}$ (μM)
**1**	30.20 ± 1.43
**2**	39.38 ± 1.44
**Cisplatin**	24.45 ± 1.03

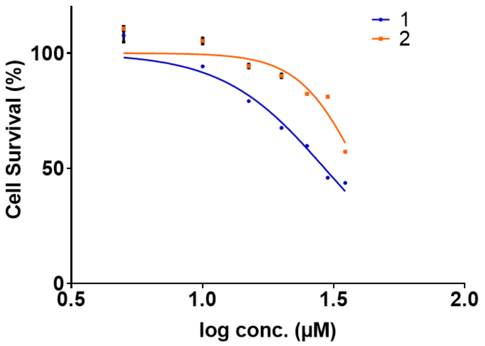

Figure S20: The percentage cell survival of MDA-MB-231 breast cancer cells as determined by MTT assays. The cancer cells were treated with either 0.1% DMSO (vehicle control), the test compounds (**1** and **2**) at concentrations between 5 μM and 35 μM. * = $p \leq 0.05$, thus statistically significant and ** = $p \leq 0.01$, thus very statistically significant.

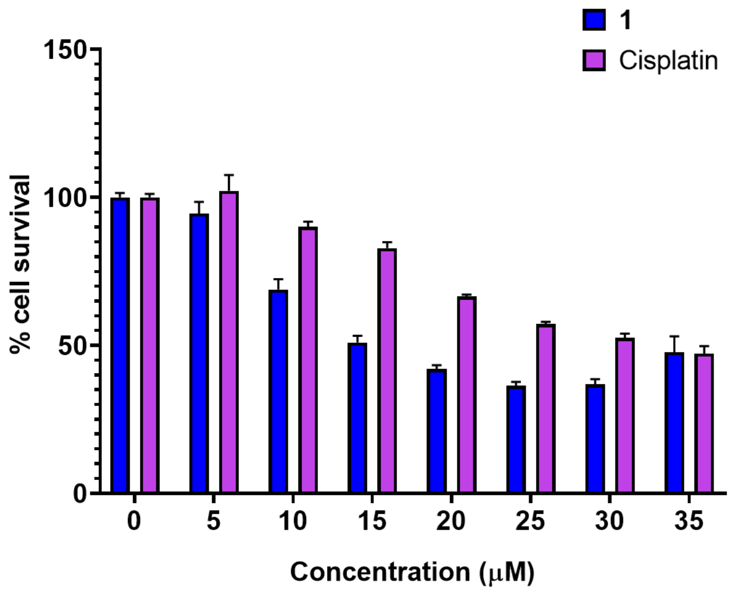

Compound	IC$_{50}$ (μM)
**1**	19.28 ± 1.16
**Cisplatin**	31.08 ± 1.044

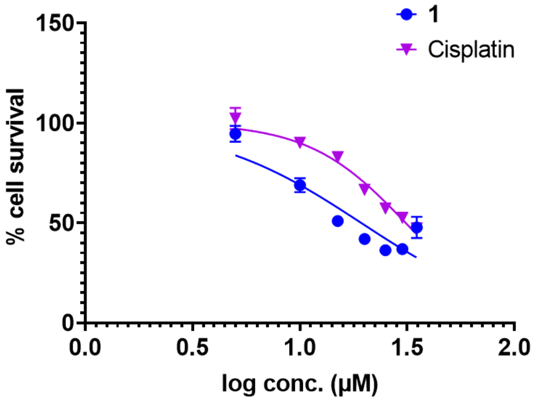

Figure S21: The percentage cell survival of PANC-1 pancreatic cancer cells as determined by MTT assays. The cancer cells were treated with either 0.1% DMSO (vehicle control), the test compounds (**1** and **2**) at concentrations between 5 μM and 35 μM. * = $p \leq 0.05$, thus statistically significant and ** = $p \leq 0.01$, thus very statistically significant.

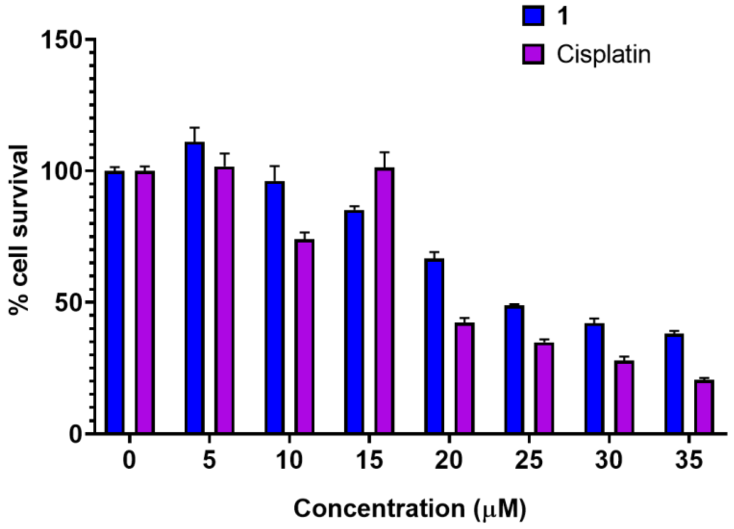

Compound	IC_{50} (μM)
**1**	26.71 ± 1.05
**Cisplatin**	18.85 ± 1.07

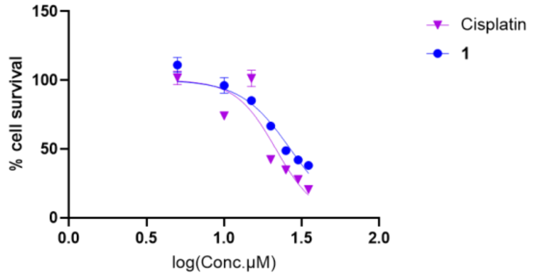

Figure S22: The percentage cell survival of CFPAC-1 pancreatic cancer cells as determined by MTT assays. The cancer cells were treated with either 0.1% DMSO (vehicle control), the test compounds (**1** and **2**) at concentrations between 5 μM and 35 μM. * = $p \leq 0.05$, thus statistically significant and ** = $p \leq 0.01$, thus very statistically significant.

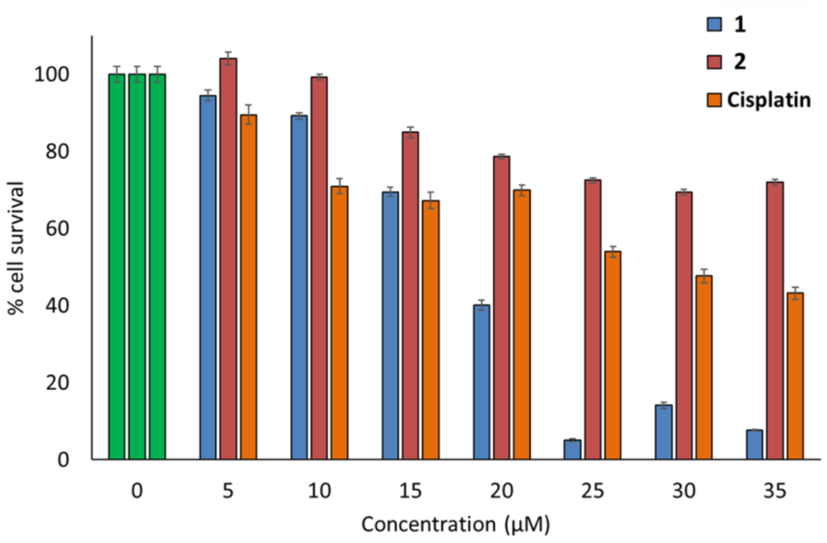

Compound	IC_{50} (μM)
**1**	17.64 ± 0.59
**2**	54.17 ± 4.02*
**Cisplatin**	29.51 ± 1.46

* IC_{50} value extrapolated by Graphpad Prism V8.0.2

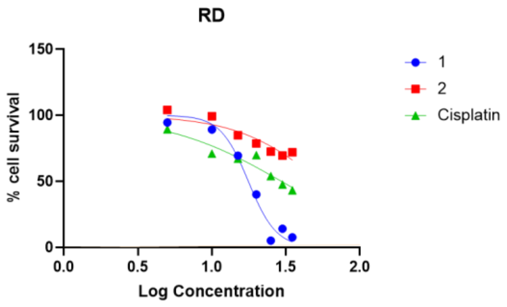

Figure S23: The percentage cell survival of RD rhabdomyosarcoma cells as determined by MTT assays. The cancer cells were treated with either 0.1% DMSO (vehicle control), the test compounds (**1** and **2**) at concentrations between 5 μM and 35 μM. * = $p \leq 0.05$, thus statistically significant and ** = $p \leq 0.01$, thus very statistically significant.

Compound	IC_{50} (μM)
**1**	17.02 ± 0.68
**2**	50.77 ± 2.89*
Cisplatin	36.73 ± 1.03

* IC_{50} value extrapolated by Graphpad Prism V8.0.2

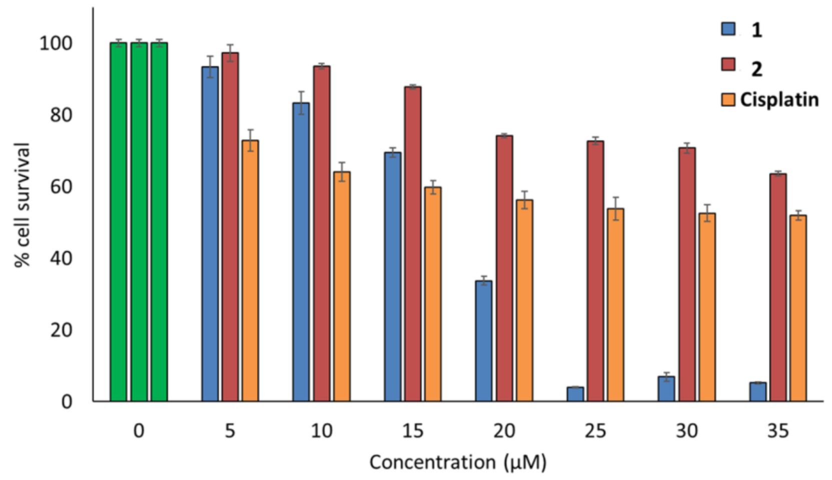

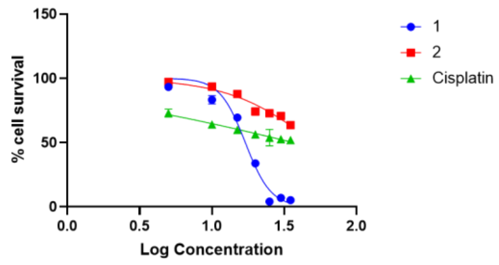

Figure S24: The percentage cell survival of RH-30 rhabdomyosarcoma cells as determined by MTT assays. The cancer cells were treated with either 0.1% DMSO (vehicle control), the test compounds (**1** and **2**) at concentrations between 5 μM and 35 μM. * = p ≤ 0.05, thus statistically significant and ** = p ≤ 0.01, thus very statistically significant.

Compound	IC_{50} (μM)
**1**	36.19 ± 1.05*
**2**	48.88 ± 1.15*
Cisplatin	43.52 ± 1.08*

* IC_{50} value extrapolated by Graphpad Prism V8.0.2

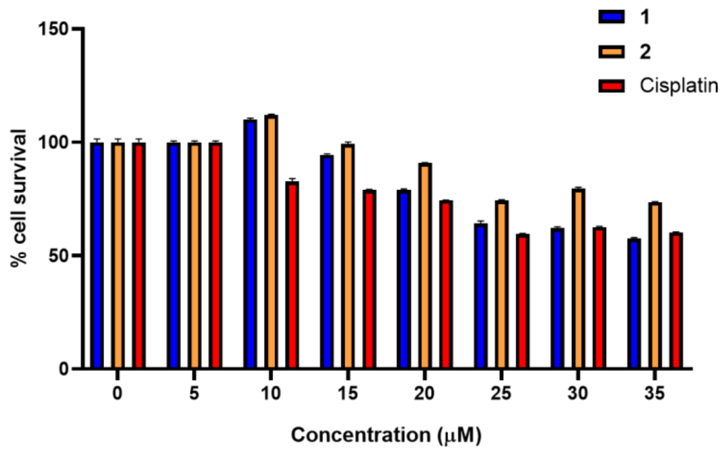

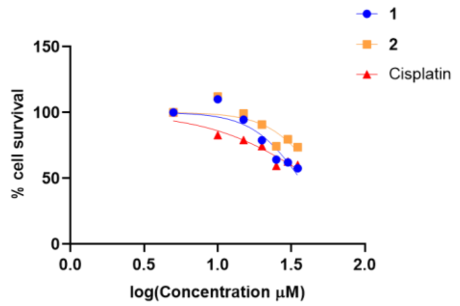

Figure S25: The percentage cell survival of FG-0 non-tumorigenic cells as determined by MTT assays. The cancer cells were treated with either 0.1% DMSO (vehicle control), the test compounds (**1** and **2**) at concentrations between 5 μM and 35 μM. * = p ≤ 0.05, thus statistically significant and ** = p ≤ 0.01, thus very statistically significant.

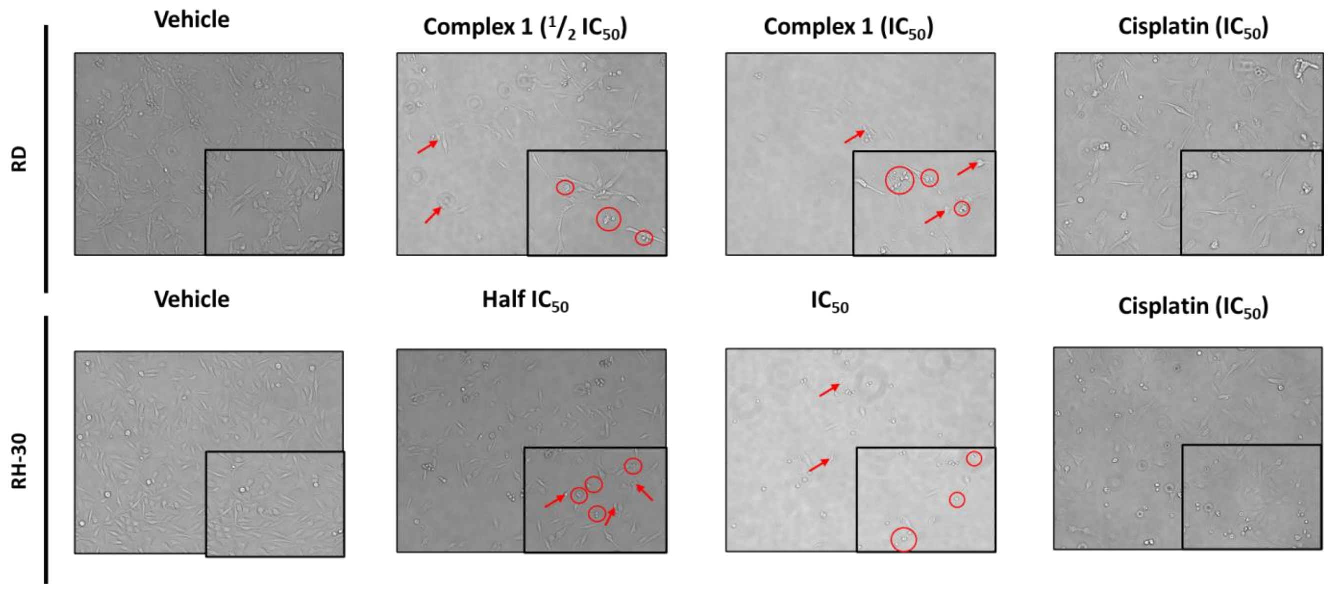

Figure S26: Representative images (10X; EVOS M5000 imaging System) of the morphology of RD and RH-30 cells after treatment with either the complex **1** (at ½ IC_{50} or IC_{50}) or cisplatin for 48 h.

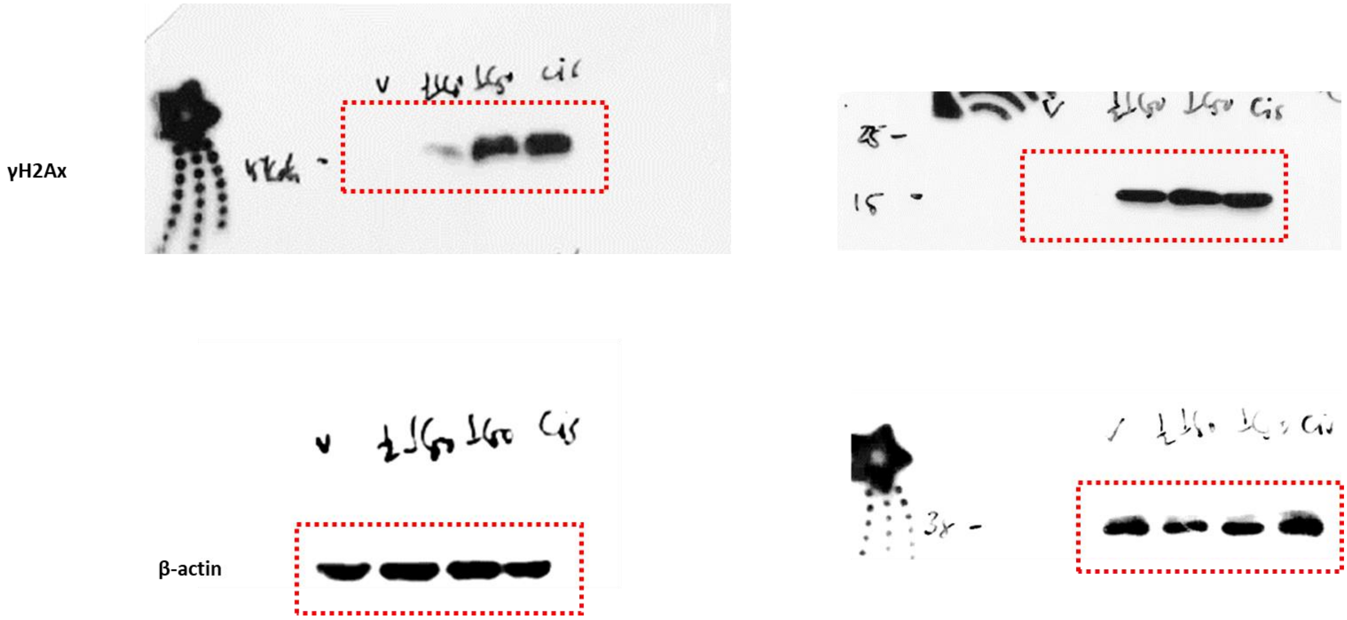

Figure S27: The uncropped Western blot in Figure 5a.

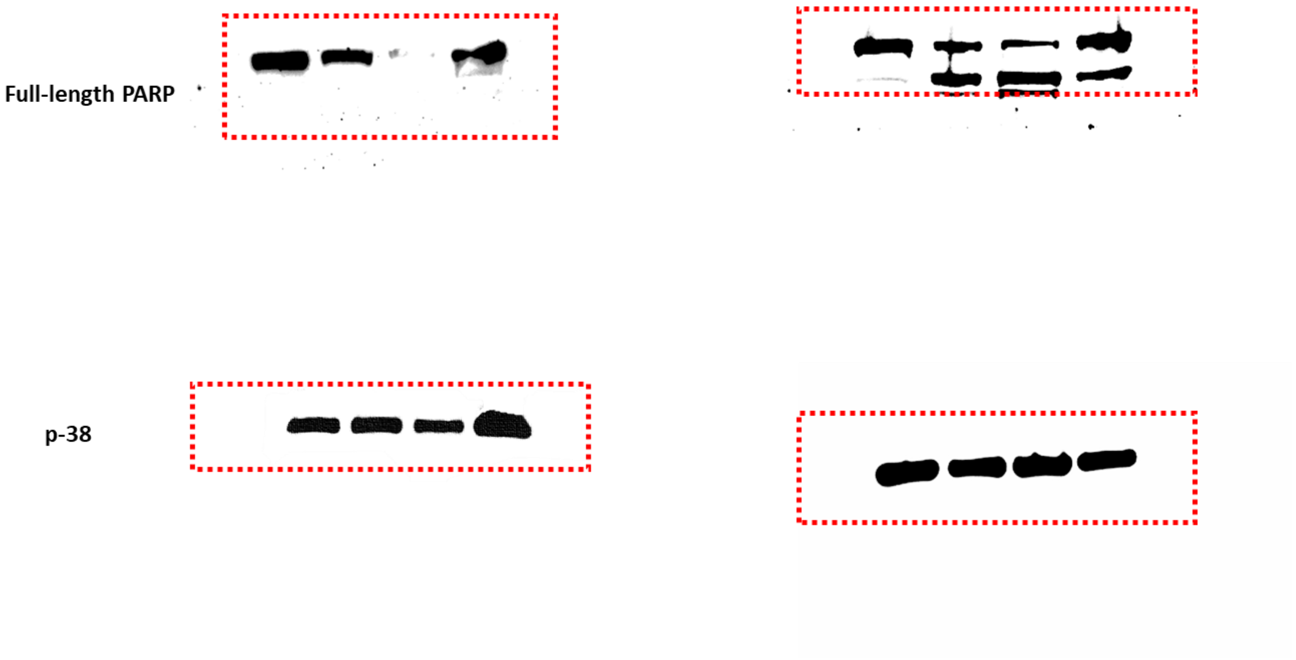

Figure S28: The uncropped Western blot in Figure 5b.

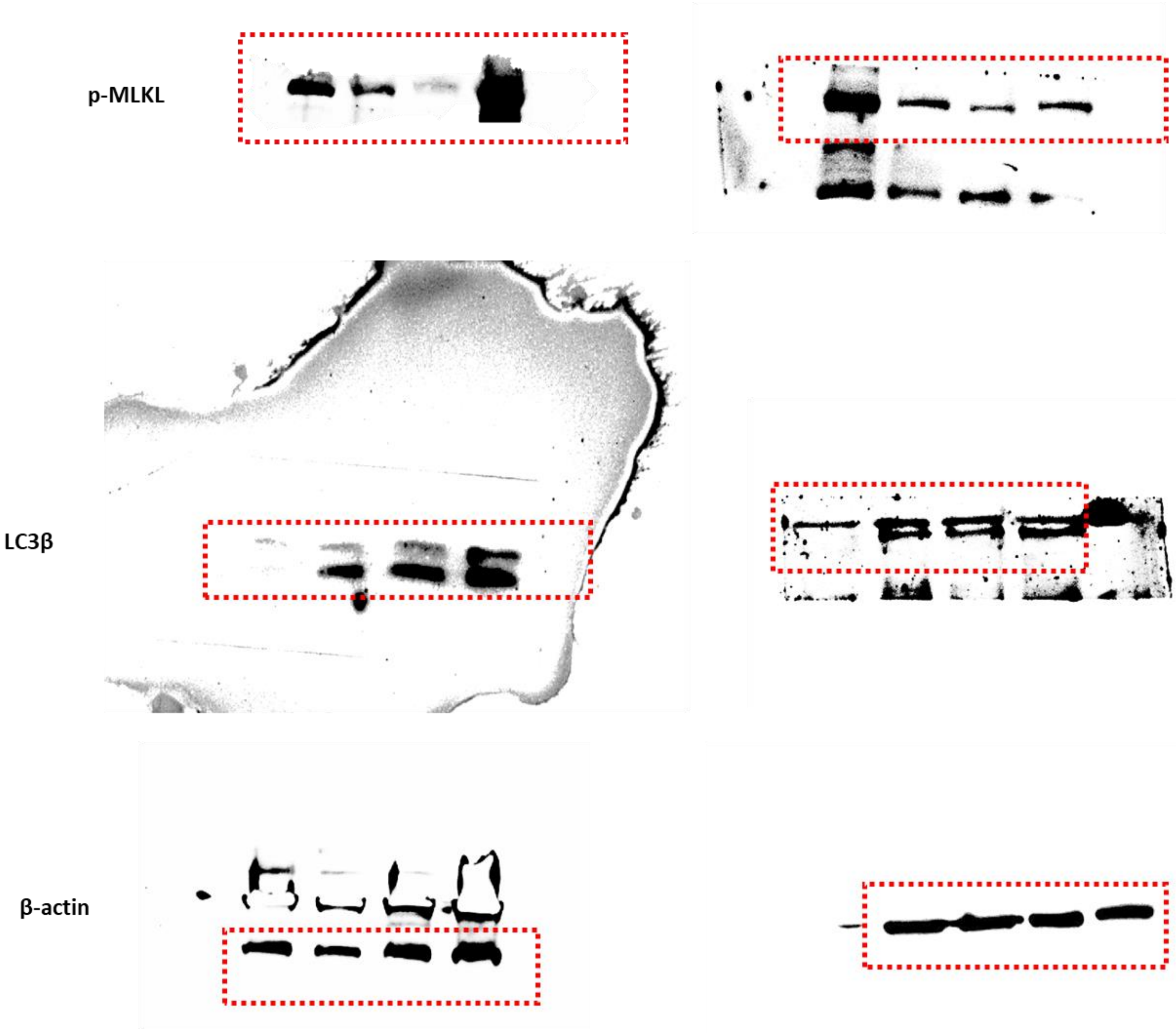

Figure S29: The uncropped Western blot in Figure 5b.

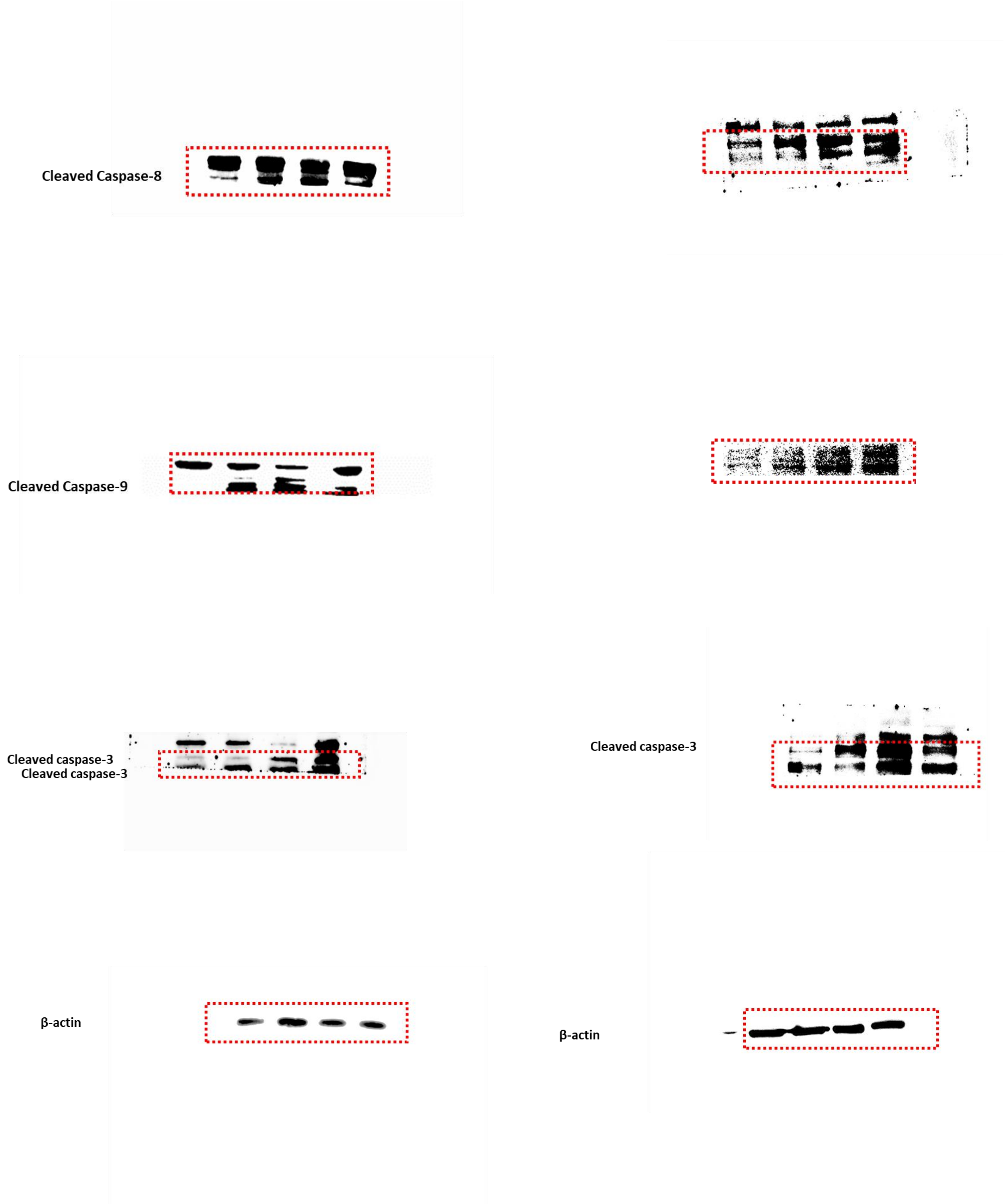

Figure S30: The uncropped Western blot in Figure 6.

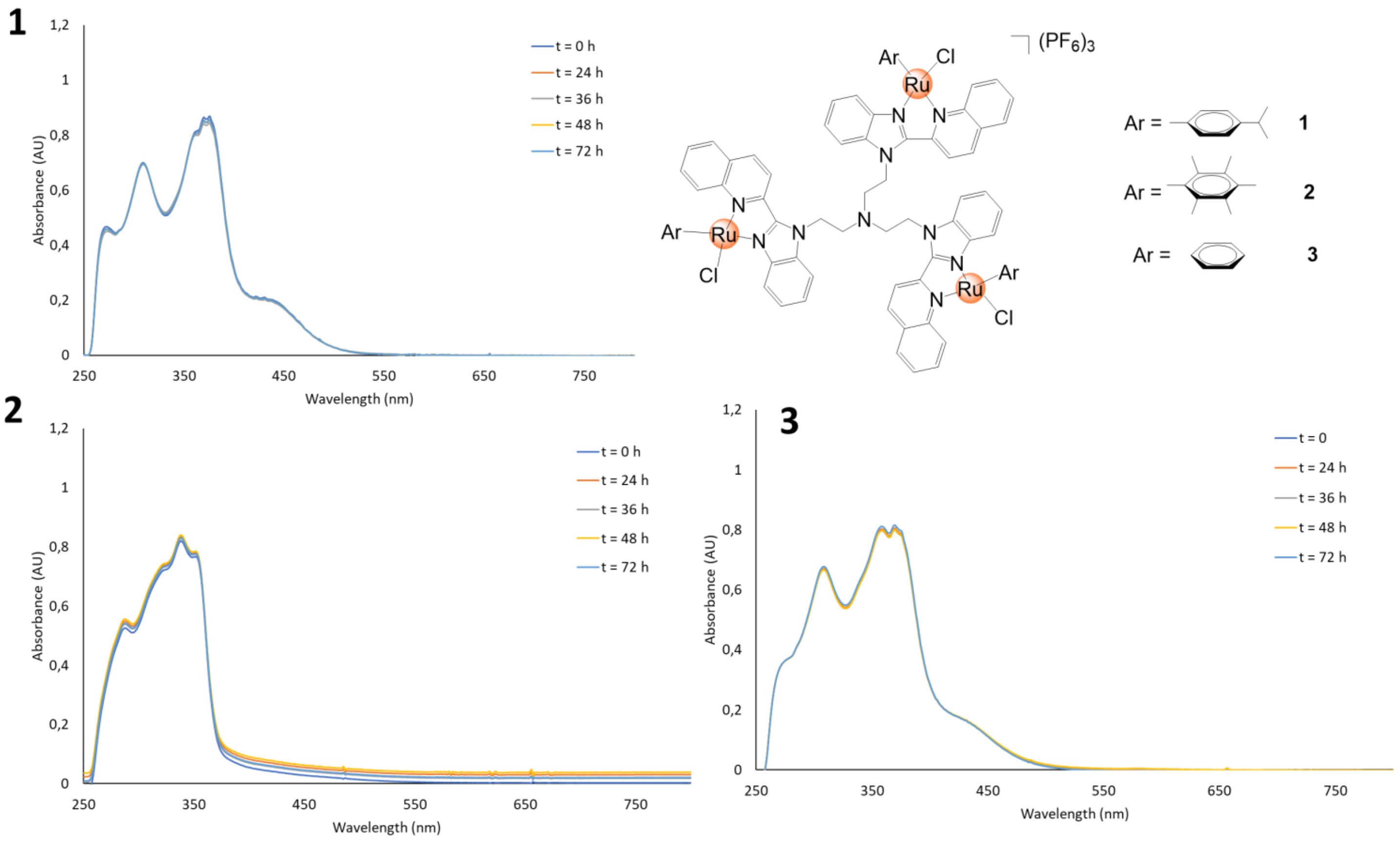

Figure S31: The UV/ Vis spectra of the complexes **1** – **3** (at 10 µM) in 100% DMSO maintained at 37 °C.
